# Impact of Lepidopteran Oral Secretions on the Transcriptome of *Arabidopsis thaliana*

**DOI:** 10.1002/pld3.70085

**Published:** 2025-06-19

**Authors:** Angel Fernandez Martin, Philippe Reymond

**Affiliations:** ^1^ Department of Plant Molecular Biology University of Lausanne Lausanne Switzerland

**Keywords:** Arabidopsis, glucosinolates, herbivory, lepidoptera, oral secretions, transcriptome, wound healing, wounding

## Abstract

Plants respond to attack by chewing insects through the recognition of herbivore‐associated molecular patterns (HAMPs) that are present in oral secretions (OS) and released at the wound site, leading to appropriate deployment of plant immune responses. Because insect feeding is accompanied by severe wounding of the leaf tissue, the specific contribution of HAMPs to defense is not well characterized. Also, OS contain effectors that interfere with the activation of defenses, but the underlying downregulated genes are poorly studied. Here, we analyzed the transcriptome of *Arabidopsis thaliana* leaves in response to wounding alone or to wounding and application of OS from *Spodoptera littoralis* or *Pieris brassicae* . For both insects, OS amplified wound‐induced responses and specifically promoted the activation of stress and hormonal pathways, as well as pathogen‐related responses. In contrast, OS inhibited the expression of genes involved in the regulation and biosynthesis of aliphatic glucosinolates (GS), and cell wall strengthening. In addition, OS‐mediated suppression of wound‐induced *ERF114* and wound healing‐related genes uncovered a novel strategy to impair defenses. In support of these findings, we observed an increased performance of *S. littoralis* and *P. brassicae* larvae feeding on OS‐treated Arabidopsis plants. Altogether, we highlight a major contribution of OS components to plant response to herbivory and unveil the potential role of conserved OS‐derived effector(s) in inhibiting defenses.

## 1 Introduction

Due to their sessile nature, plants have evolved a high degree of transcriptional plasticity that allows them to quickly adapt their metabolism to environmental changes. Upon the perception of wounding, a transcriptional cascade is induced within minutes and extends for over 24 h (Moore et al. 2022). Plant immune responses to wounding and herbivory are initiated by the perception by cell‐surface receptor kinases of damage‐associated molecular patterns (DAMPs) and herbivore‐associated molecular patterns (HAMPs), followed by a series of downstream signaling steps including phosphorylation of the receptors and associated factors, activation of specific ion channels and production of reactive oxygen species (ROS) (Erb and Reymond 2019; Li et al. 2020). These signals are integrated by hormonal pathways and result in transcriptional changes leading to the deployment of specific anti‐herbivore immune responses. Jasmonic acid (JA) is the main pathway involved in the regulation of such changes but other phytohormones, such as salicylic acid (SA), ethylene, auxin, gibberellin, and abscisic acid (ABA), have been shown to be involved to different extents in the response to herbivory (Erb and Reymond 2019). There is also evidence for crosstalk between these hormonal pathways (Pieterse et al. 2012). Following wounding or attack by chewing herbivores, polyunsaturated fatty acids from the chloroplast membrane are enzymatically converted into JA, that is in turn conjugated to isoleucine, forming JA‐Ile, the major bioactive component of this pathway. JA‐Ile mediates the interaction of JAZ repressors with SCF^COI1^complex, leading to the ubiquitination and degradation of JAZs, followed by activation of MYCs, which are basic helix–loop–helix (bHLH) transcription factors that act as a central regulatory hub for JA‐dependent defenses (Kazan and Manners 2013; Howe et al. 2018). In Arabidopsis, MYC2, MYC3, and MYC4 act additively to regulate a substantial fraction of JA‐dependent transcriptional changes and contribute significantly to defense against herbivores (Schweizer, Fernández‐Calvo, et al. 2013). Arabidopsis constitutively produces a basal level of the defense compounds glucosinolates (GS). Upon herbivory, the production of GS is further enhanced through MYC‐dependent activation of the expression of GS biosynthesis genes. However, this process requires direct binding of MYC2/3/4 with MYB transcription factors (Schweizer, Fernández‐Calvo, et al. 2013). Indeed, MYB28, MYB29, and MYB76 regulate the transcription of methionine‐derived aliphatic GS biosynthesis genes, whereas MYB34, MYB51, and MYB122 are necessary for the biosynthesis of tryptophan‐derived indole GS (Mitreiter and Gigolashvili 2021). Like MYC2/3/4, these MYBs are crucial for Arabidopsis defense against leaf‐chewing herbivores. Indeed, *myb28/29* double mutant is impaired in the production of aliphatic GS and is more susceptible to lepidopteran herbivores (Beekwilder et al. 2008; Müller et al. 2010), whereas overexpression of *MYB51* increases indole‐GS accumulation and leads to reduced herbivory by *Spodoptera exigua* (Gigolashvili et al. 2007).

Lepidopteran OS is a complex mixture of molecules including elicitors, effectors, and digestive enzymes. The substantial enzymatic activity of OS is necessary for the insect to digest plant macromolecules and structures, in order to facilitate ingestion and access to plant nutriments (Prajapati et al. 2024). This enzymatic activity, however, causes the release of DAMPs that amplify plant immune responses (Li et al. 2020). In Arabidopsis, lipases contained in OS of the grasshopper *Schistocerca gregaria* cause accumulation of OPDA, which leads to an increase of JA and subsequently to a generalized induction of JA‐dependent responses (Schäfer et al. 2011). OS of the stink bug *Nezara virdula* contain a large set of active enzymes involved in cell wall digestion, triggering immune responses in soybean (Giacometti et al. 2020). In addition, HAMPs in caterpillars OS induce plant defense responses through activation of the JA pathway (Wu and Baldwin 2009). The receptor‐like protein INR of *Vigna unguiculata* has recently been shown to perceive inceptin peptides derived from *Spodoptera furgiperda* OS and constitutes the first HAMP receptor identified (Steinbrenner et al. 2020).

On the other hand, OS‐derived effectors inhibit plant immune responses through different mechanisms (Snoeck et al. 2022; Prajapati et al. 2024). For chewing herbivores, HARP1 and HAS1 are proteinaceous effectors in OS of the cotton bollworm *Helicoverpa armigera*. HARP1 stabilizes JAZs repressors, interfering with the release of MYC transcription factors whereas HAS1 directly binds to MYCs and prevents their activity (Chen et al. 2019; Chen et al. 2023). In Arabidopsis, unknown compounds in OS of the chewing lepidopteran herbivores *Pieris brassicae* and *Spodoptera littoralis* inhibit the expression of wound‐responsive genes, leading to a better insect performance (Consales et al. 2012).

Transcriptome analysis on plants infested by herbivores has greatly contributed to the understanding of transcriptional regulation of anti‐herbivore responses. However, such studies have focused mainly in the identification on plant transcriptional signatures specific for the type of insect herbivore, rather than studying the contribution of herbivore‐derived compounds on plant transcriptional changes (Coolen et al. 2016; Garcia et al. 2021; Pamplona et al. 2022; Montesinos et al. 2024). Application of OS to mechanical wounds has been shown to faithfully, although not completely, simulate natural herbivory (Bricchi et al. 2010; Lortzing et al. 2017). However, it allows to standardize the temporal and spatial responses to herbivory and permits to separate mechanical from biotic components of the stimulus. Few studies have assessed the contribution of herbivore‐derived compounds to wound‐induced transcriptional responses. A whole‐genome study on inceptin‐treated cowpea leaves showed that this HAMP amplifies wound transcriptional responses and induce terpene synthases and peroxidases involved in volatile production (Steinbrenner et al. 2022). A microarray gene expression profiling comparing poplar transcriptional changes induced by wounding and by OS of the caterpillar *Malacosoma disstria* showed that OS induces a transcriptional reprogramming qualitatively similar to the one caused by wounding (Major and Constabel 2006). A similar study on the expression of ca. 5500 genes from the gymnosperm *Picea stichenisis* revealed a considerable overlap between wounding and application of OS from two different insect pests (Ralph et al. 2006). In maize, a transcriptome analysis of mechanical wounding and simulated herbivory by *Mythimna separata* OS showed that the latter elicited a stronger and longer‐lasting response (Qi et al. 2016).

In Arabidopsis, research investigating the expression of defense‐related genes in response to wounding and herbivory has partially shown the contribution of herbivore‐derived components to wound‐induced transcriptional responses. However, this contribution has never been studied at a whole genome level (Reymond et al. 2000). Here, we thus tested the specific role of OS on whole‐genome transcriptional responses by performing an RNA sequencing on Arabidopsis leaves that were wounded or treated with OS from the specialist *P. brassicae* or the generalist *S. littoralis* .

## 2 Results

### 2.1 OS Application has a Stronger Effect on Gene Expression Than Wounding

**FIGURE 1:**
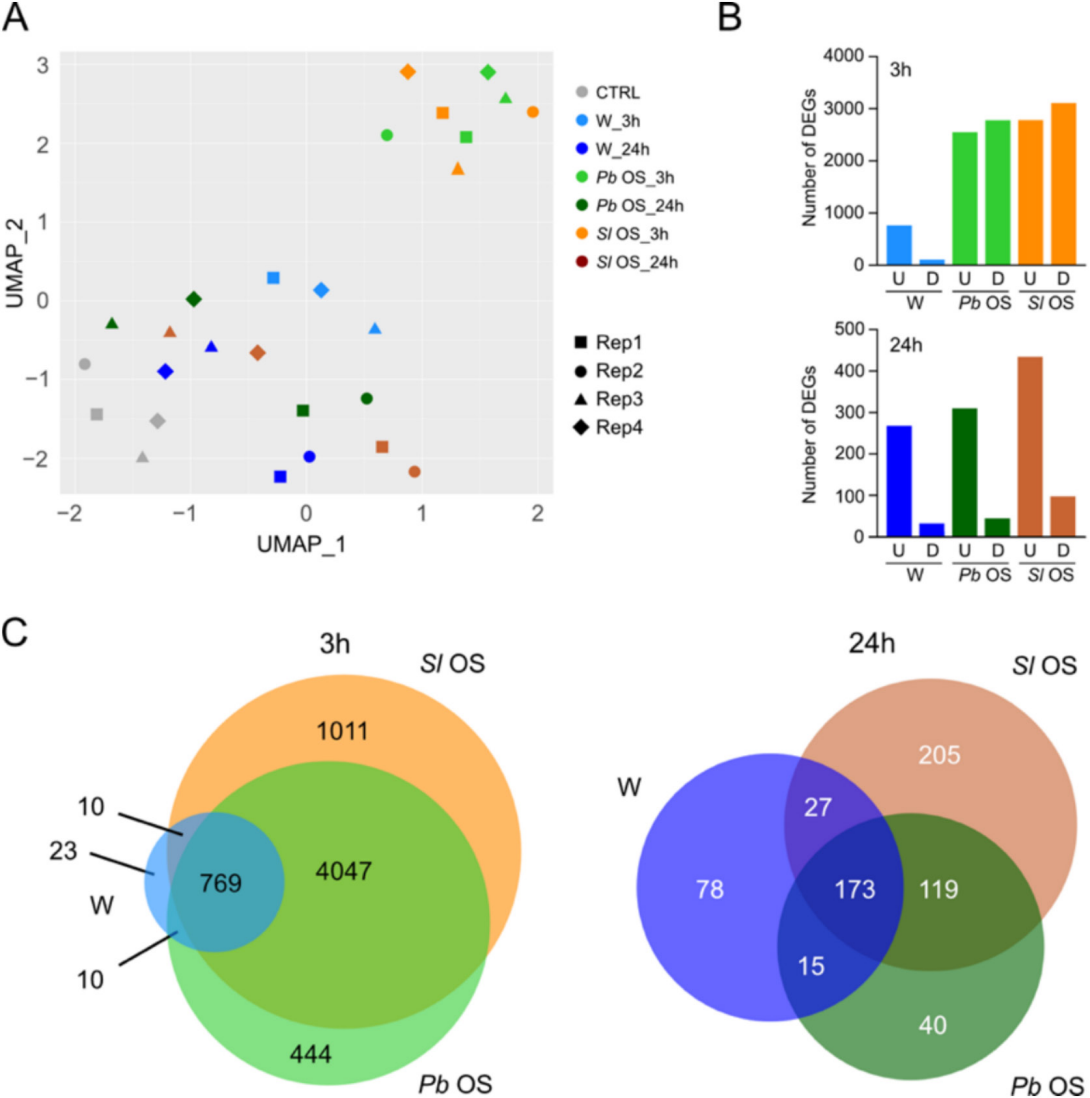
Number of DEGs through treatments. (A) Uniform manifold approximation and projection (UMAP) analysis of the global expression profile data. Symbols of the same color represent different independent biological replicates. Plants were wounded and treated with H_2_O (W), oral secretions from ***P. brassicae*** (*Pb* OS), or oral secretions from *Spodoptera littoralis* (*Sl* OS) for 3 h or 24 h. CTRL, unwounded plants. (B) Number of differentially regulated genes (DEGs) (log_2_FC > 1 or < −1, adj*P* < 0.05) in plants subjected to different treatments. U, upregulated; D, downregulated. (C) Number of overlapping and unique DEGs between treatments.

To study the transcriptional responses of Arabidopsis to wounding and herbivore OS, we performed RNA sequencing (RNA‐seq) on unwounded leaves, leaves wounded and treated with water, and leaves wounded and treated with either *P. brassicae* or *S. littoralis* OS. We analyzed four independent biological replicates collected 3 h and 24 h after treatments. By piercing four 1 mm hole in three leaves, we generated a relatively moderate wounding that may mimic the effect of feeding by a few neonate/early instar larvae. The rationale for choosing 3 h was that the expression of JA‐dependent genes and JA accumulation peak between 2 and 4 h (Reymond et al. 2000). In addition, the 24 h time‐point might allow to detect late responses. To assess the patterns of variation within the data, UMAP analysis was performed on the RNA‐Seq data (Figure 1A). The 3 h samples treated with *P. brassicae* and *S. littoralis* OS clustered, suggesting that the two OS treatments have similar effects on the transcriptomic response of the plant. At 24 h, all treatments tended to cluster together, including the unwounded control, indicating that most transcriptional changes induced by wounding and OS returned to basal expression levels (Figure 1A). The same can be observed in volcano plots depicting fold change of gene expression between wounded and unwounded treatments (Figure S1).

Looking at the number of differentially expressed genes (DEGs) after each treatment, wounding and OS induced significant transcriptional changes at 3 h and this drastically diminished at 24 h. In wounded leaves, a total of 812 DEGs were found at 3 h, with 734 upregulated and 78 downregulated genes. At 24 h, the number of DEGs decreased to 293, with 264 upregulated and 29 downregulated. In OS‐treated leaves, a much larger number of DEGs was found. *P. brassicae* OS triggered 5270 DEGs at 3 h, with 2520 upregulated and 2750 downregulated, while this number decreased to 347 DEGs at 24 h, with 306 upregulated and 41 downregulated genes. *S. littoralis* OS triggered 5837 DEGs at 3 h, with 2754 upregulated and 3083 downregulated, followed by a decrease to 524 DEGs at 24 h, with 430 upregulated and 94 downregulated (Figure 1B). Despite the large difference in the number of DEGs between wounding and OS treatment, it is interesting to note that most genes differentially expressed upon wounding were also differentially expressed when plants were treated with OS. Indeed, there were only 23 genes/5270 (*P. b*.) or 23/5837 (*S. l*.) that were differentially regulated by wounding but not by any of the OS at 3 h. This proportion was slightly larger at 24 h (78/347 (*P. b*.) or 78/524 (*S. l*.)) (Figure 1C). This indicates that, overall, treatment with OS has a weak qualitative effect on the transcriptional response triggered by wounding alone.

The transcriptional responses to OS treatments were very similar, with 81% and 73% of shared up‐ and downregulated genes, respectively. Thus, the two lepidopteran species, despite one being a generalist and the other a specialist, trigger very similar transcriptional responses. However, at 24 h, OS of the two different species shared 57% of upregulated genes and 19% of downregulated genes, suggesting that in the long‐term different compositions of the OS may have a different impact on the transcriptional changes they induce on the host (Figure 1C).

**FIGURE 2:**
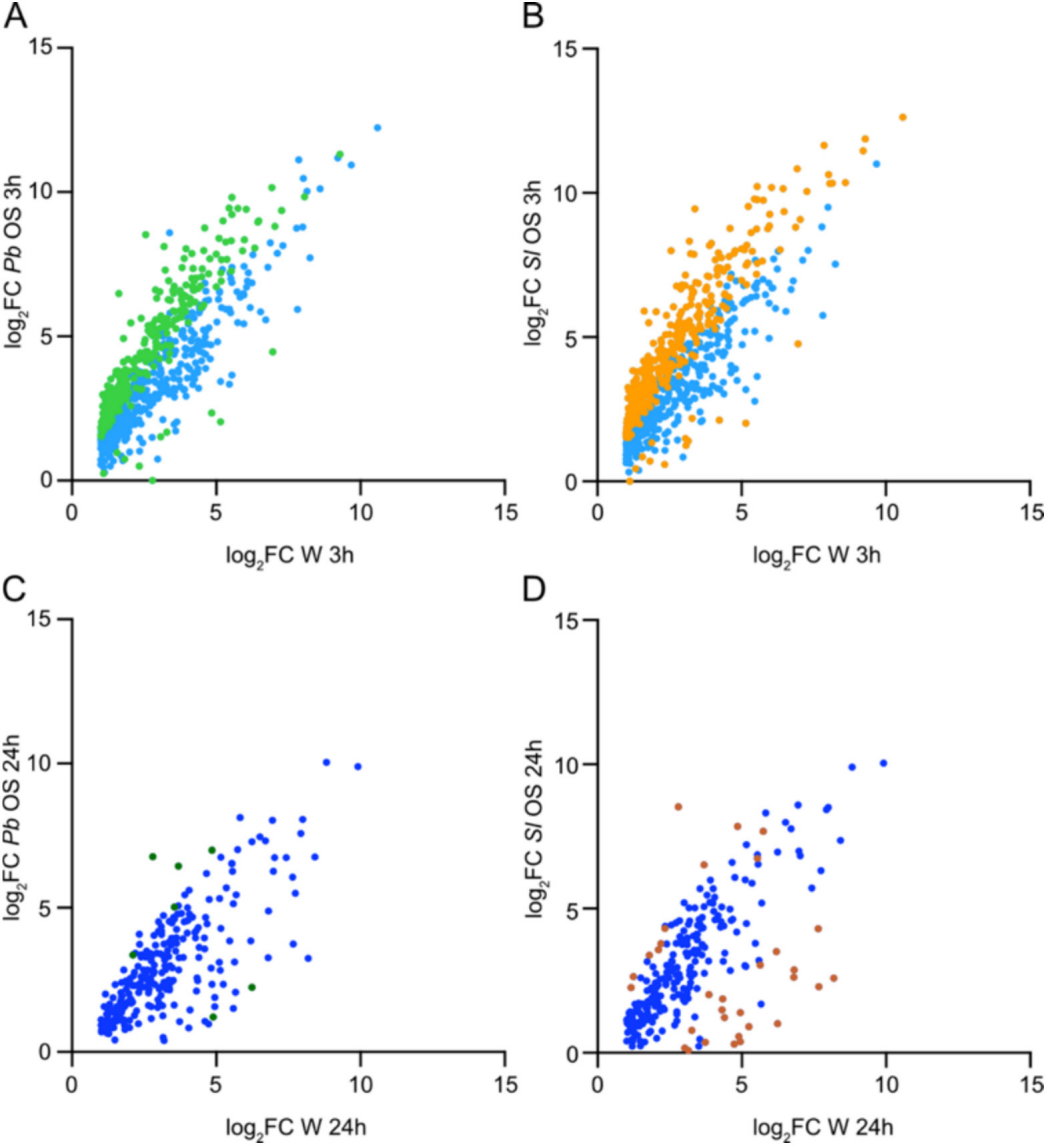
OS effect on wound‐induced genes. Expression of genes significantly induced by wounding (W) (log_2_FC > 1, adj*P* < 0.05) after 3 h (A, B) and 24 h (C, D) is shown and compared to treatment with ***P. brassicae*** OS (A, C) or ***S. littoralis*** OS (B, D). Light and dark blue dots represent genes equally induced between W and OS treatments. Light and dark green or orange dots represent genes significantly more or less induced by OS treatments than by W.

When looking at the amplitude of induction, a substantial number of wound‐responsive genes were significantly more induced 3 h after *P. brassicae* OS (280/734) or *S. littoralis* OS (325/734) application, whereas only few genes were significantly less induced (10/734 for *P. brassicae* and 13/734 for *S. littoralis* ) (Figure 2A,B; Table S1). At 24 h, this amplification was no longer present, with only a few genes being more induced by OS (Figure 2C,D; Table S1). Thus, the addition of OS clearly amplifies the early wound response at the transcriptional level. When looking at downregulated genes, the contribution of OS to wounding is also illustrated by genes that are significantly more repressed 3 h, while this amplification is not present at 24 h (Figure S2; Table S1).

### 2.2 Specificity of the Transcriptional Response to OS

**FIGURE 3:**
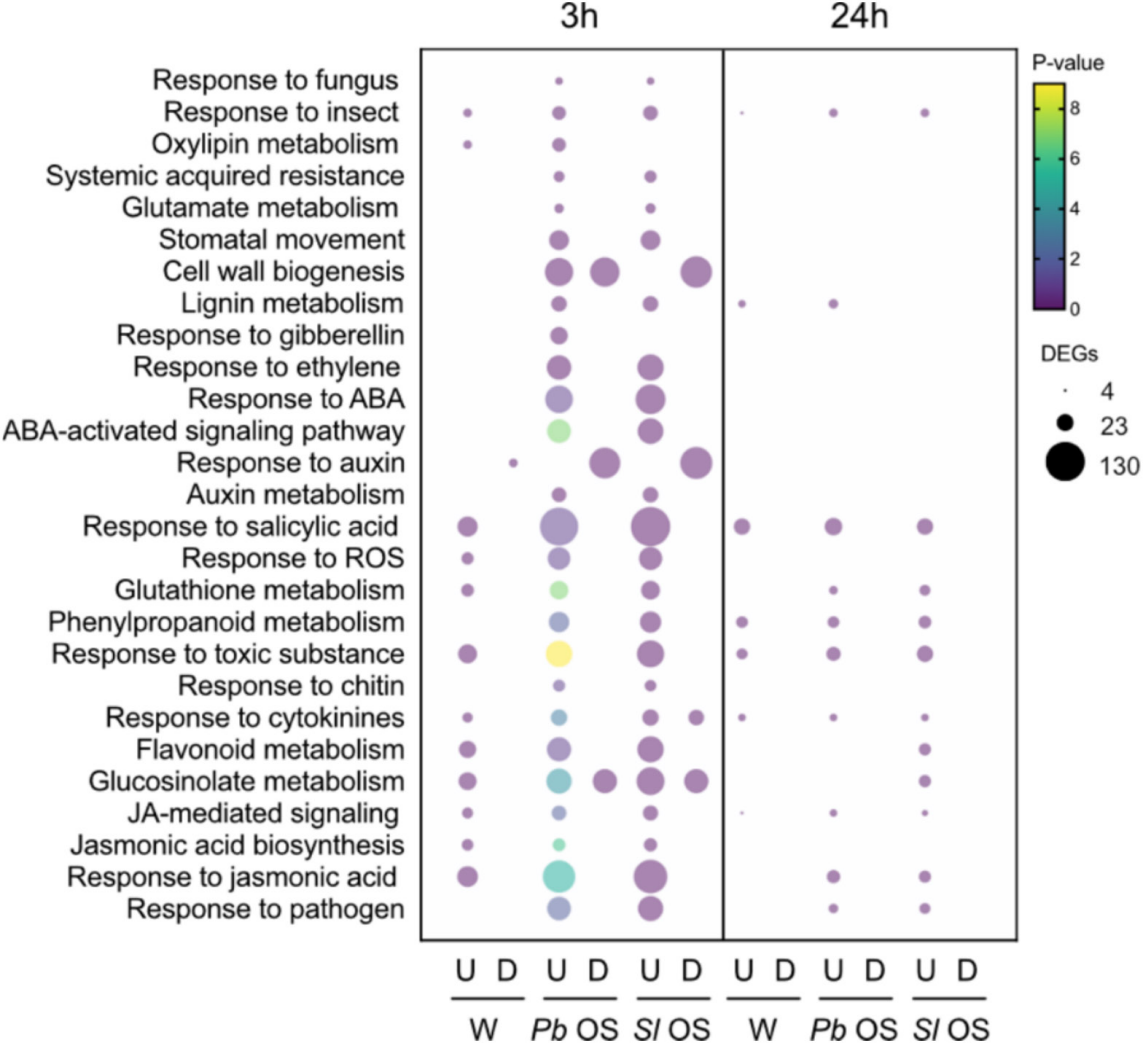
Enrichment analysis of differentially expressed genes (DEGs) between treatments. Immune‐related GO terms for DEGs (log_2_FC > 1, adj*P* < 0.05) are grouped by biological function. Bubble sizes represent the number of DEGs associated to each term. Colors represent the *p*‐value adjusted by Bonferroni correction. U, upregulated; D, downregulated.

To have an overview of the biological processes regulated by wounding or OS treatment, we performed an enrichment analysis of up‐ and downregulated genes based on their Gene Ontology (GO) and we plotted GO terms related to immune responses (Figure 3). At 3 h, DEGs from wounded plants were enriched in GO terms including the JA pathway, the oxylipin pathway, GS biosynthesis, cytokinin and flavonoid metabolism, and ROS responses. Interestingly, GO terms like “response to SA” and “response to auxin” were found in up‐ and downregulated genes, respectively, which could indicate that some wound‐induced genes participate in the JA‐SA and JA‐auxin crosstalk. OS treatment led to the upregulation of a larger number of genes included in the same GO terms enriched in wounded plants, confirming that OS‐derived HAMPs exacerbate the defense response triggered by wounding (Figure 3). Furthermore, OS specifically induced the expression of genes belonging to GO terms associated with hormonal pathways, such as ET, ABA, GA, and auxin. OS also triggered the upregulation of genes associated with GO terms “response to chitin”, “response to fungus”, “response to pathogen”, and “systemic acquired resistance”, suggesting the presence of bacteria and/or fungi, as previously reported in OS of other chewing insects (Chung et al. 2013).

**FIGURE 4:**
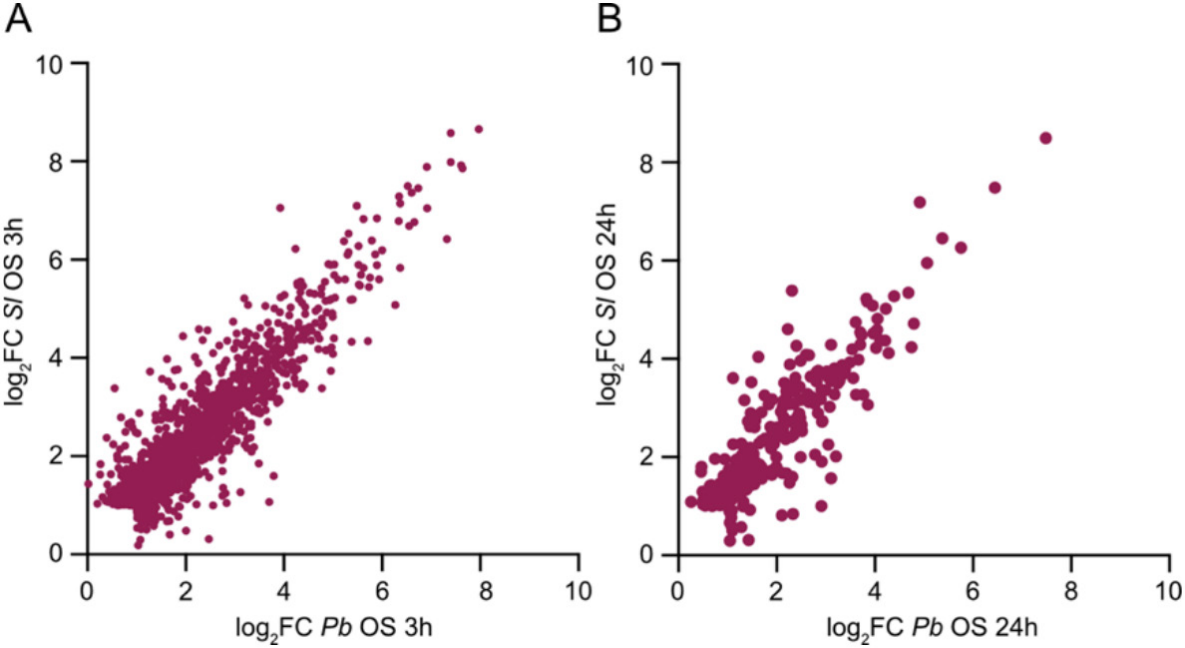
Specific gene upregulation by ***P. brassicae*** and ***S. littoralis*** OS. Genes significantly induced by OS (log_2_FC > 1, adj*P* < 0.05) and not induced by wounding (log_2_FC < 1 or log_2_FC > 1 but adj*P* > 0.05) after 3 h (A) and 24 h (B) are shown.

Like with the global comparison of DEGs (Figures 1 and 2), the analysis of genes specifically induced 3 h or 24 h after *P. brassicae* and *S. littoralis* OS application showed that both OS trigger a highly similar profile (Figure 4, Table S1). The same is true for downregulated genes (Figure S3). Strikingly, this suggests that HAMPs from these two divergent herbivorous species are quite similar or that OS of different composition converge on a conserved transcriptional activation.

To evaluate if the transcriptome of plants exposed to simulated herbivory by wounding and OS application reflects the response to natural herbivory, we compared our data with a previous study from our group where Arabidopsis plants were challenged with larvae of *S. littoralis* for 5 h and global expression was analyzed with DNA microarrays (Schweizer, Bodenhausen, et al. 2013). Although sensitivity, dynamic range and specificity of RNAseq is much higher, we found that a large proportion of genes induced by *S. littoralis* OS application for 3 h are also induced by larvae (Figure S4; Table S2).

**FIGURE 5:**
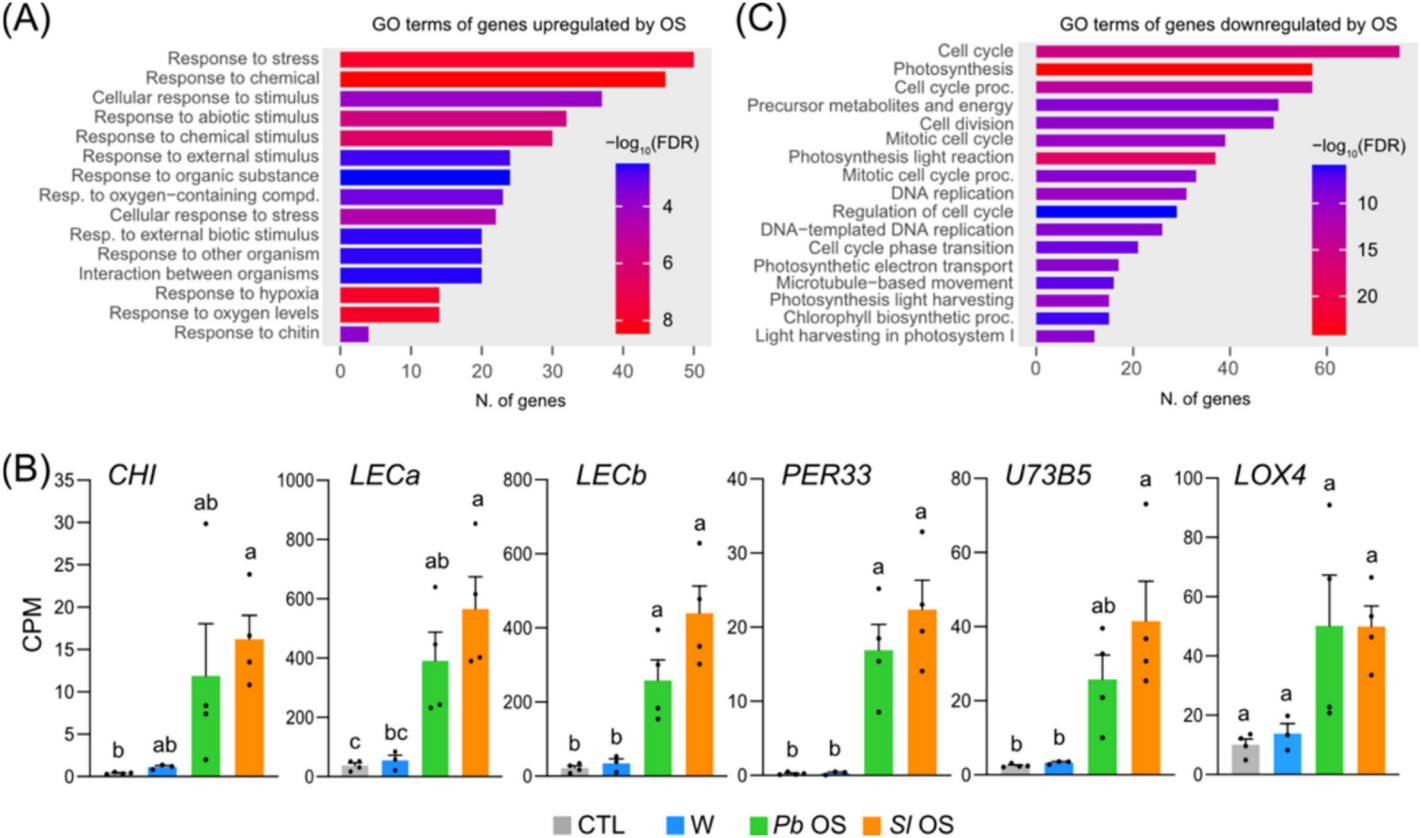
Specific effects of insect OS on Arabidopsis transcriptome. An enrichment analysis of gene ontology (GO) categories was performed for genes whose expression is specifically induced (A) or repressed (B) at 3 h by ***P. brassicae*** and ***S. littoralis*** OS, and not by wounding, using a threshold for differential expression of log_2_FC > 2 or log_2_FC < −2. (C) Relative expression of selected genes specifically induced by OS. Bars represent means ± SE of four biological replicates. Different letters represent significant differences at ***p*** < 0.05 (ANOVA, followed by Tukey HSD for multiple comparisons). CPM, counts per million of reads. *CHI* (At2g43570), chitinase; *LECa* (At3g16530)/*LECb* (At3g15356), lectin‐like; *PER33* (At3g49110), peroxidase; *U73B5* (*UGT73B5*) (At2g15480), UDP‐glycosyltransferase, *LOX4* (At1g72520), lipoxygenase. CTL, unwounded; W, wounding; *Pb* OS, treatment with ***P. brassicae*** OS; *Sl* OS, treatment with *Spodoptera littoralis* OS.

To further understand the specific contribution of OS components, we assessed the GO term of genes that were strongly up‐ or downregulated (log_2_FC > 2 or log_2_FC < −2) after OS treatment but not after wounding. For upregulated genes, GO terms involved in defense processes were found, including responses to stress, biotic stimulus, interaction between organisms, response to chitin, response to external stimulus, and response to oxidative stress (Figure 5A). In particular, OS specifically induced *CHI*, a chitinase, *LECa*, and *LECb*, two lectin‐like that are induced by chitin elicitors (Lyou et al. 2009) (Figure 5B). This observation suggests that plants perceive chitin of insect origin or derived from symbiotic fungi present in the digestive tract of insects. Also, upregulation of *PER33*, a peroxidase involved in ROS production during pathogenesis (Daudi et al. 2012) and *UGT73B5*, a glycosyltransferase linked with ROS production in resistance to *Pseudomonas syringae* (Simon et al. 2014), highlights a potential role of OS‐derived microbes in triggering immune responses (Figure 5B). Of potential interest, the trend for OS‐mediated induction of *LOX4*, one of the four 13‐LOX genes involved in JA biosynthesis, supports the known herbivore‐mediated enhancement of JA accumulation (Qi et al. 2016) but suggests a specific role for this homolog that would deserve further testing (Figure 5B). For downregulated genes, the majority were enriched in the GO terms “cell cycle”, “cell division,” and “photosynthesis”, confirming the known growth‐defense tradeoffs observed in response to biotic stresses (Bilgin et al. 2010; Guo et al. 2018) (Figure 5C).

In summary, the important transcriptomic changes observed after OS application reveal that the response to chewing herbivores has a prominent biotic contribution from OS that leads to the amplification of the wound response and a specific activation or inhibition of additional pathways.

### 2.3 OS Downregulate the Expression of GS Biosynthetic Genes and Genes Involved in Cell Wall Processes

The induction of defense genes in response to herbivory or OS application in different plants and in response to diverse arthropods has been the focus of numerous studies. In contrast, analysis of the downregulation of defense genes has received much less attention, although OS are known to contain effectors that impair defenses (Stahl et al. 2018). We thus specifically explored our dataset to identify such process.

First, the rationale for performing a transcriptome analysis on samples collected 3 h after treatment was to monitor the impact of OS on early wound responses, such as the induction of the JA pathway. Given that OS effectors have been shown to target the JA pathway (Chen et al. 2019; Chen et al. 2023), we hypothesized that expression of JA‐related genes might also be affected. However, we found that, overall, genes involved in JA biosynthesis or signaling were similarly induced by wounding or OS treatments (Figure S5). Thus, it appears that OS derived from the two divergent lepidopteran herbivores do not compromise the JA pathway at the transcriptional level in Arabidopsis.

**FIGURE 6:**
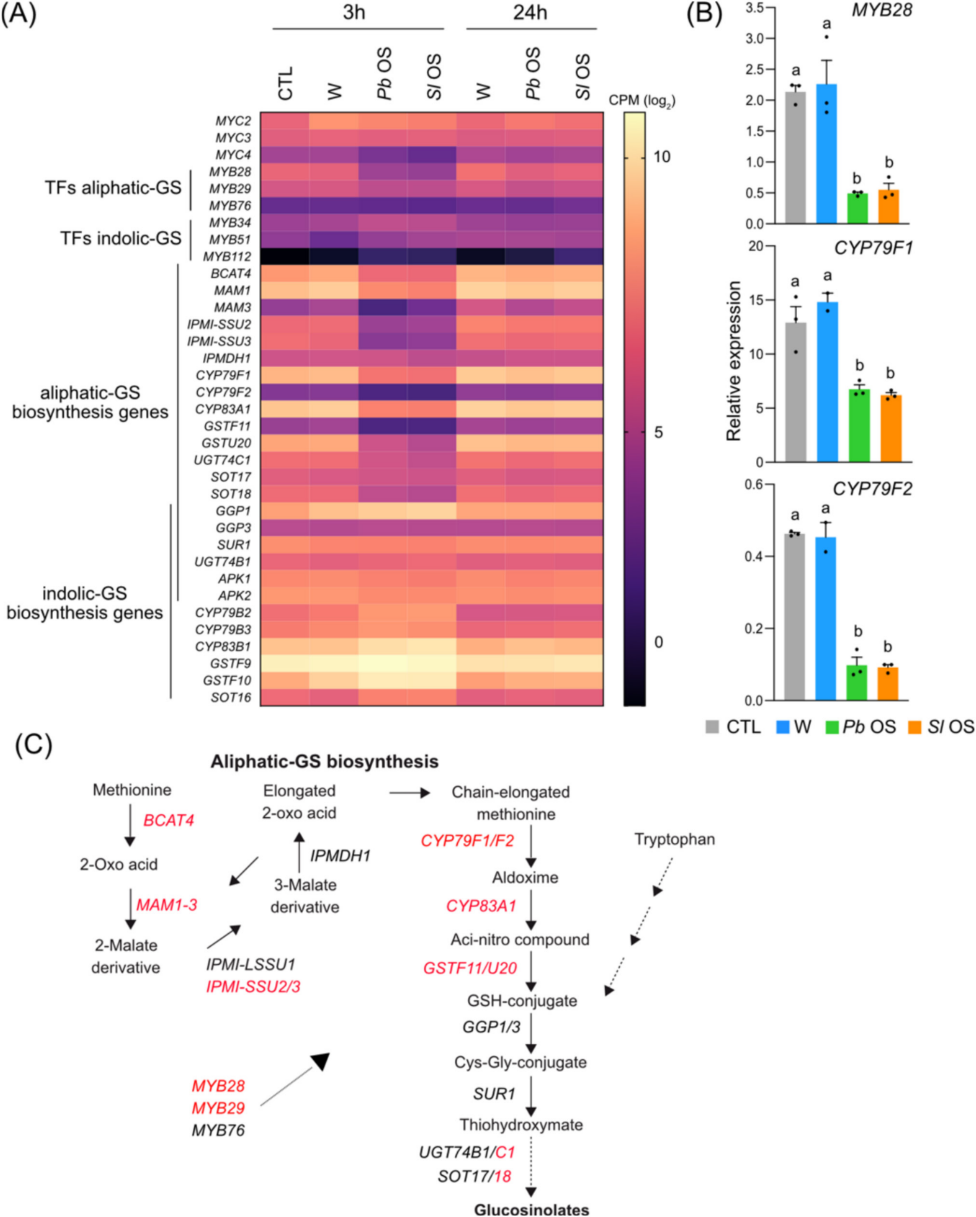
Oral secretions (OS) downregulate genes involved in the biosynthesis of aliphatic glucosinolates (GS). (A) Heatmap showing the expression profile of genes involved in the regulation and biosynthesis of GS. (B) Expression level of *MYB28* (At5g61420), *CYP79F1* (At1g16410), and *CYP79F2* (At1g16400) 3 h after treatment. Transcript levels were monitored by qPCR. Relative expression (normalized to the housekeeping gene *SAND* (At2g28390)) represents mean ± SE of three technical replicates. The experiments were repeated three times with similar results. Different letters represent significant differences at ***p*** < 0.05 (ANOVA, followed by Tukey HSD for multiple comparisons). (C) GS biosynthesis pathway. Genes involved in the biosynthesis of aliphatic‐GS are displayed while the indole‐GS branch starting with tryptophan is only indicated. Genes in red are suppressed by both OS at 3 h. MYB28/29/76 transcription factors are known to regulate the aliphatic‐GS branch (thick dashed arrow). CPM, counts per millions of reads; TFs, transcription factors.

Expression of GS biosynthetic genes plays a central role in protecting Arabidopsis against polyphagous herbivores such as *S. littoralis* . Mutants in the biosynthesis of these defense compounds have been shown to be more vulnerable to *S. littoralis* herbivory (Schweizer, Fernández‐Calvo, et al. 2013). Besides being major components of induced defenses, GS are also part of the constitutive defenses of *Brassicaceae* plants (Mitreiter and Gigolashvili 2021). Indeed, genes involved in their biosynthesis are constitutively expressed in Arabidopsis, under the regulation of MYC and MYB transcription factors. MYC2, MYC3, and MYC4 have been shown to directly interact with MYB factors in order to regulate the transcription of indole and aliphatic GS biosynthetic genes (Schweizer, Fernández‐Calvo, et al. 2013). Strikingly, OS of both caterpillars suppressed the basal expression of *MYC4*, *MYB28*, and *MYB29* and most genes responsible for aliphatic GS biosynthesis (Figure 6A), three of which were further validated by qPCR (Figure 6B). This finding contrasts with the induction of a subset of GS genes that were enriched in wounded and OS‐treated samples (Figure 3). However, these genes primarily belong to the biosynthesis of indole‐GS and to GS modification enzymes (Table S1). Thus, considering that MYB28/29/76 are specific regulators of aliphatic‐GS biosynthesis, OS‐dependent decreased expression of these factors might explain the specific reduced expression of aliphatic‐GS biosynthesis genes (Figure 6C). In addition, suppression of *MYC4* expression might also contribute to a lower activity of MYBs. Since aliphatic GS are the most abundant GS in Arabidopsis (Petersen et al. 2002), an early OS‐mediated suppression of their biosynthesis might antagonize the induction of indole‐GS and overall provide a significant advantage to *S. littoralis* development.

**FIGURE 7:**
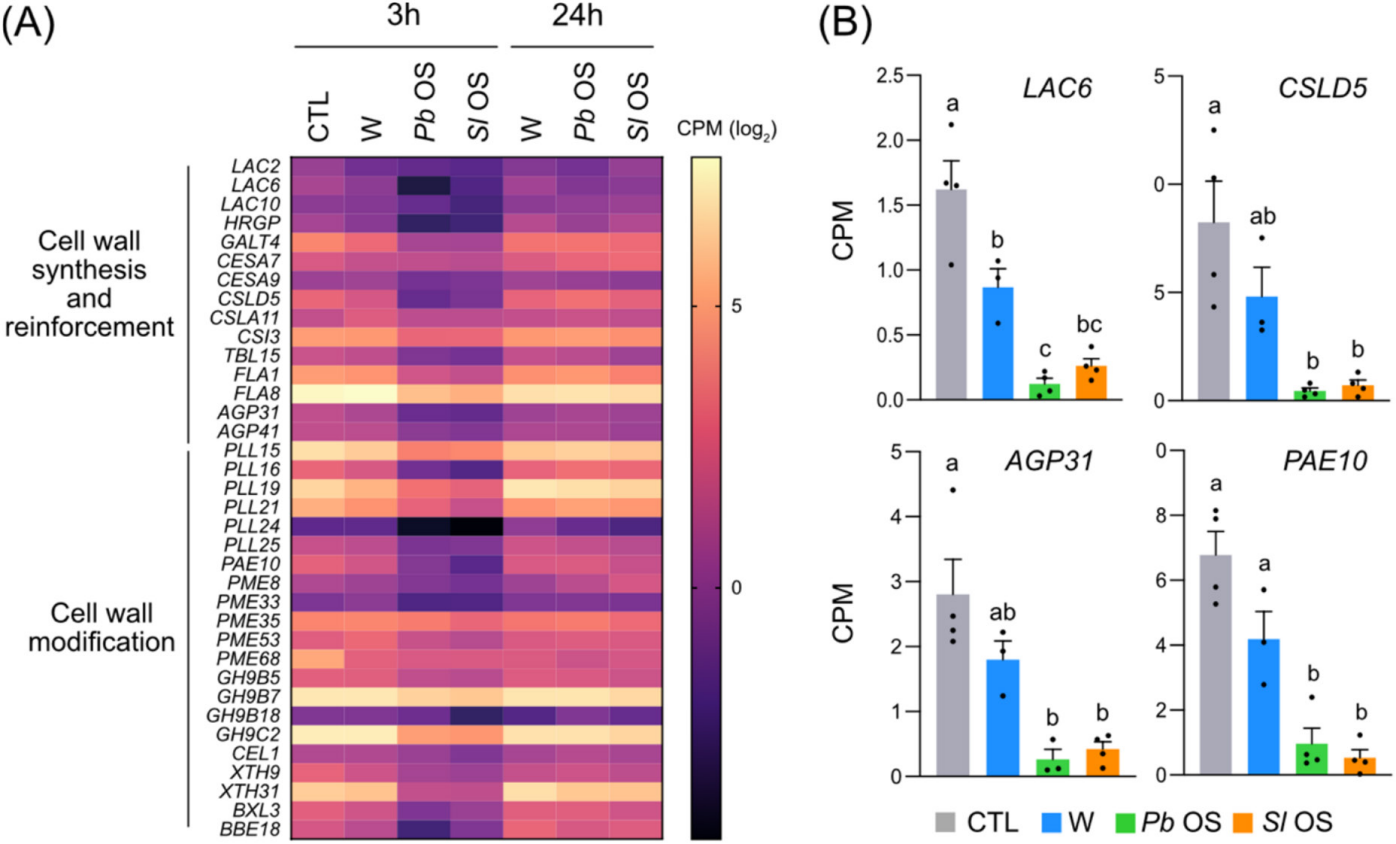
OS‐mediated downregulation of genes involved in cell wall processes. (A) Heatmap showing the expression profile of genes related to cell wall biogenesis and modification. (B) Expression of laccase 6 (*LAC6*, At2g46570), cellulose synthase (*CSLD5*, At1g02730), arabinogalactan protein (*AGP31*, At1g28290) and pectin acetylesterase 10 (*PAE10*, At5g26670). Values represent mean ± SE of four biological replicates. Different letters represent significant differences at ***p*** < 0.05 (ANOVA, followed by Tukey HSD for multiple comparisons). CPM, counts per millions of reads.

Cell wall composition, metabolism, and modifications are known to participate in plant defenses, in particular against herbivores (Lucas 2000; Hanley et al. 2007; Whitney and Federle 2013; Molina et al. 2024). Upon herbivory, cell walls act as physical defense barriers and their damage leads to a release of DAMPs, such as oligogalacturonides, that trigger plant immune responses. Additionally, increased cellulose and lignin synthesis, pectin modification, cross‐linking between cell wall proteins and components lead to cell wall strengthening. For instance, increased lignin accumulation in rice protects against feeding by the brown planthopper (He et al. 2020). Interestingly, the expression of genes involved in cell wall synthesis and remodeling was downregulated 3 h after OS application (Figure 7A). Among these, there were laccases (including *LAC6*, Figure 7B) that play a role in lignin synthesis by oxidative coupling of monolignol precursors, a few cellulose synthases (including *CSLD5*, Figure 7B), hydroxyproline‐rich glycoproteins, arabinogalactan proteins (including *AGP31*, Figure 7B) that participate in cell wall strengthening by creating supramolecular scaffolds with cell wall polysaccharides (Hijazi et al. 2014), pectin modifying enzymes (including the pectin acetyl esterase *PAE10*, Figure 7B), a member of the TRICHOME BIREFRINGEN‐LIKE family (*TBL15*) that O‐acetylates polysaccharide thus increasing herbivore resistance (Sun et al. 2020), cellulases, xyloglucan hydrolases, and a xylosidase (Figure 7A). Thus, during feeding, OS release by chewing herbivores may substantially inhibit cell wall reinforcement.

### 2.4 Suppression of Wound‐Induced Genes

We then reasoned that OS might suppress the expression of wound‐induced genes, as an additional way to interfere with the activation of defenses. Focusing on genes that were induced more than two‐fold after wounding and significantly suppressed with the addition of OS, we found a relatively small list that included several cell wall‐related genes, the herbivore‐responsive *ML3* gene and *ERF114*, which was the only gene that was suppressed at both 3 and 24 h time points (Table 1). The suppression of wound‐induced laccase (*LAC5*), dirigent protein (*DIR23*), pectate‐lyase (*PLL18*), and two cell wall proteins (At2g41800, At2g41810) at 24 h (Table 1) confirms our observation of a downregulation of cell wall‐related processes (Figure 7).

**TABLE 1:** Wound‐induced genes significantly suppressed by ***P. brassicae*** and ***S. littoralis*** OS. Expression ratios (±SE) are the average of four independent biological replicates.

AGI code	Description	Wounding	*P.b.* OS	*S.l.* OS
		log_2_FC (3 h)		
**At5g61890**	**Ethylene response factor ERF114**	**6.96**	**4.46** **	**4.77** *
At4g38410	Dehydrin family protein	5.15	2.04*	2.01**
At3g27660	Oleosin 4 OLE4	4.84	2.34*	3.5
At5g05965	Unknown	4.23	3.72	2.12*
At5g12420	Wax ester synthase WSD1	3.29	1.68**	2.18*
At5g23820	Herbivory‐induced response regulator ML3	3.15	2.10	1.40**
At1g14250	GDA1/CD39 nucleoside phosphatase	3.08	1.52*	1.24**
At4g18440	Adenylosuccinate lyase	2.34	0.51**	0.58**
At3g28060	Nodulin MtN21‐like transporter	2.79	0.01*	2.10
At5g02940	Chloroplast envelope ion channel PEC1	1.88	1.50	1.34*
At2g32487	Unknown	1.82	0.75*	0.70**
At1g31550	Esterase/acyltransferase/lipase	1.56	0.97*	0.85**
At3g15790	Methyl‐CpG‐binding protein MBD11	1.33	0.50	0.44*
At5g48850	Sulfate deficiency‐induced gene SDI1	1.32	−0.53*	−0.31*
At2g22170	Lipase/lipoxygenase PLAT2	1.13	0.28	−0.05**
At3g55940	Phospholipase C7 PLC7	1.10	0.25*	0.32
		**log**_**2**_ **FC (24 h)**		
At5g09530	Proline‐rich protein PELPK1	8.19	3.25	2.58*
At3g22620	Plasma membrane‐anchored lipid‐transfer protein LTPG20	7.67	3.74	2.29**
At2g41800	Cell wall protein TEB	7.65	6.06	4.30*
At3g02885	Flowering regulator GASA5	6.81	4.89	2.88**
At2g41810	Cell wall protein/pectin metabolism	6.81	3.27	2.61**
At2g40370	Laccase LAC5	6.24	2.24*	1.01**
**At5g61890**	**Ethylene response factor ERF114**	**6.20**	**3.85**	**3.51** *
At4g03540	CASP‐like protein	5.64	3.12	3.04*
At3g55090	ABCG16 transporter	5.25	2.35	0.90**
At2g48130	Plasma membrane‐anchored lipid‐transfer protein LTPG15	4.95	1.57	0.38**
At3g44550	Fatty acid reductase FAR5	4.95	1.89	1.39*
At4g28110	ABA‐responsive protein MYB41	4.90	1.21*	0.57**
At5g41040	Suberin feruloyl‐transferase RWP1	4.73	0.97	0.30*
At3g50400	Esterase/acyltransferase/lipase	4.61	1.07	0.20**
At2g20825	Developmental regulator ULTRAPETALA2	4.40	3.27	1.23**
At3g25640	Unknown	4.33	2.57	1.87*
At2g39350	ABCG1 transporter	4.32	2.11	1.49*
At5g13580	ABCG6 transporter	4.04	0.83	−0.05**
At3g27400	Pectate‐lyase PLL18	3.86	3.27	2.01*
At2g21100	Dirigent protein DIR23	3.73	1.05	0.37*
At1g03820	Unknown	3.27	2.17	0.77*
At2g38530	Lipid‐transfer protein LTP2	3.16	0.50	−0.27**
At1g06520	Glycerol‐phosphate acyltransferase GPAT1	3.15	1.25	0.07**
At4g39320	Microtubule‐associated protein	3.02	1.69	0.16**

* *p* < 0.05.

** *p* < 0.01.

Interestingly, *ML3* is a member of the ML (MD‐2‐related lipid recognition) gene family. ML proteins enhance innate immunity in mammals by interacting with Toll‐like receptors (Park et al. 2009). *ML3* expression was shown to be regulated by wounding and JA and a *ml3* mutant was more susceptible to *S. littoralis* herbivory, indicating that *ML3* is involved in herbivore‐mediated responses (Fridborg et al. 2013; Hakenjos et al. 2013). Thus, the suppression of *ML3* expression during feeding by an OS‐derived effector may constitute an efficient strategy to attenuate defenses. The understanding of the molecular mechanisms involved in this response clearly deserves further research.

**FIGURE 8:**
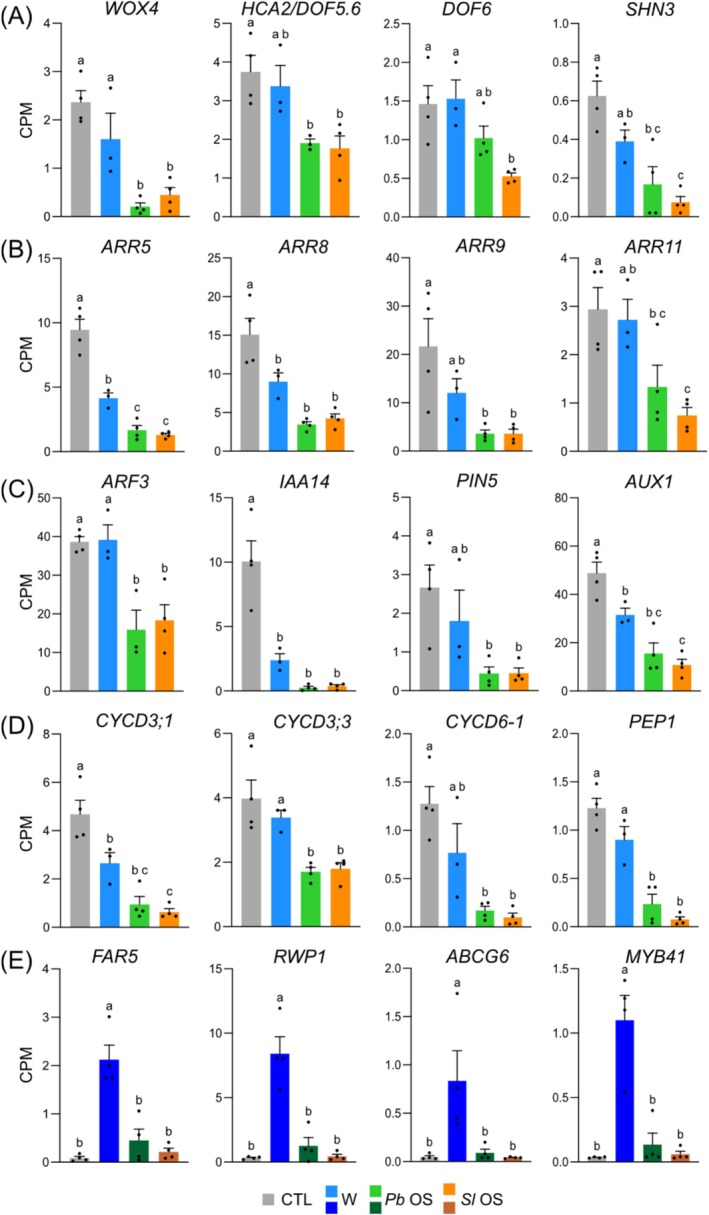
Expression of genes involved in wound healing. (A) Regulators of tissue regeneration. *WOX4* (At1g46480), *HCA2*/*DOF5.6* (At5g62940), *DOF6* (At3g45610), *SHN3* (At5g25390). (B) Cytokinin signaling genes. *ARR5* (At3g48100), *ARR8* (At2g41310), *ARR9* (At3g57040), *ARR11* (At1g67710). (C) Auxin signaling and transport genes. *ARF3* (At2g33860), *IAA14* (At4g14550), *PIN5* (At5g16530), *AUX1* (At2g38120) (D) Cyclin and peptide genes. *CYCD3;1* (At4g34160), *CYCD3;3* (At3g50070), *CYCD6‐1* (At4g03270), *PEP1* (At5g64900) (E) Suberin biosynthesis. *FAR5* (At3g44550), *RWP1* (At5g41040), *ABCG6* (At5g13580), *MYB41* (At4g28110). Values represent mean ± SE of four biological replicates. A–D (3 h), E (24 h). Different letters represent significant differences at ***p*** < 0.05 (ANOVA, followed by Tukey HSD for multiple comparisons). CPM, counts per million of reads.

We observed a robust and long‐lasting OS‐mediated suppression of the ethylene response factor *ERF114* and validated this finding independently by qPCR (Figure S6). ERF114 is an ERF/AP2 transcription factor that has been shown to regulate callus formation, tissue regeneration and organ formation (Mehrnia et al. 2013; Canher et al. 2022; Zhang et al. 2022). In response to mechanical wounding, plants initiate a complex wound healing process that includes sealing and tissue regeneration. This is of great importance to prevent dehydration and penetration of pathogens at wound sites. Several studies have identified key molecular components and hormonal pathways necessary for a rapid repair of the wound site (Ikeuchi et al. 2017; Ikeuchi et al. 2018; Ikeuchi et al. 2019; Zhou et al. 2019; Serra and Geldner 2022). We thus looked at the expression of known regulators of tissue regeneration after OS treatment (Ikeuchi et al. 2019). Interestingly, several genes involved in different wound healing steps were downregulated (Figure S7A, B). When the cell‐wall matrix is damaged, WOX, WIND, DOF, and ERF transcription factors are rapidly activated and promote tissue regeneration (Ikeuchi et al. 2019; Iwase et al. 2021; Zhang et al. 2022). OS treatment suppressed wound‐induced *ERF114* expression but not its close homologs *ERF113*, *ERF115*, and *ERF109*. *WIND1/2* were also not downregulated but the expression of two out of four crucial DOF factors (Zhang et al. 2022), *HCA2* and *DOF6*, was significantly inhibited by OS (Figure 8A, Figure S7A).

Similarly, *WOX4*, but not WOX5 that promotes regeneration in the root (Zhou et al. 2019), was downregulated (Figure 8A, Figure S7A). These findings suggest that OS‐derived effectors may only target a subset of wound healing processes.

Auxin and cytokinin play pivotal roles in plant tissue regeneration (Ikeuchi et al. 2017; Ikeuchi et al. 2019). Several cytokinin signaling type‐B ARABIDOPSIS RESPONSE REGULATORs (*ARR5/8/9/11*) and cytokinin‐dependent cyclins (*CYCD3;1*, *CYCD3;3*, *CYCD6‐1*) were downregulated (Figure 8B,D; Figure S7B). The same was true for auxin signaling and transport genes (*ARF3*, *IAA14*, *PIN5*, *AUX1*) (Figure 8C, Figure S7B).

A recent study in tomato reported the role of the elicitor peptide REF1 as a key factor in wound healing (Yang et al. 2024). Since REF1 is the homolog of PEP1, a known phytocytokine involved in Arabidopsis defense (Huffaker et al. 2006), this discovery showed that defense and tissue regeneration are part of the wound response. Intriguingly, *PEP1* expression was suppressed by OS treatments (Figure 8D), providing additional support that OS may interfere with wound healing.

Sealing of the wound site involves deposition of suberin and cuticle (Fich et al. 2016; Serra and Geldner 2022; Lewandowska et al. 2024). Expression of several genes involved in the regulation, biosynthesis, transport, and polymerization of precursors of suberin and cuticle was suppressed by OS application (Figure S7A; Table 1). This included a fatty acyl‐coenzyme A reductase *(FAR5*, Figure 8E) that is known to participate to the synthesis of saturated primary fatty alcohols that are suberin components (Domergue et al. 2010), a suberin feruloyl‐transferase (*RWP1*, Figure 8E), ABCG transporters (*ABCG6*, Figure 8E) and lipid‐transfer proteins (*LTPG15*, *LTPG20*) that export suberin precursors to the cell wall (Lee and Suh 2018; Gräfe and Schmitt 2021), the ABA‐dependent regulator of suberin synthesis *MYB41* (Figure 8E) and an ABA transporter (*ABCG16*) (Kosma et al. 2014; Zhou et al. 2024). For cuticle, downregulated genes included a member of the SHINE family of AP2/EREB TFs (*SHN3*, Figure 8A) that regulate wax biosynthesis (Aharoni et al. 2004), a lipid‐transfer protein (*LTP2*) (Jacq et al. 2017) and a wax synthase (*WSD1*). Of note, several of these genes were suppressed at 24 h (Table 1, Figure S7A), indicating that suberin and cuticle deposition is a rather late step of the wound healing process. Also, the late suppression of *LAC5* and *DIR23* (Table 1), two genes involved in lignin biosynthesis, supports the recent finding that lignin accumulation is also involved in wound healing (Xu et al. 2024).

### 2.5 OS Effectively Suppress Defenses for the Benefit of Larval Growth

**FIGURE 9:**
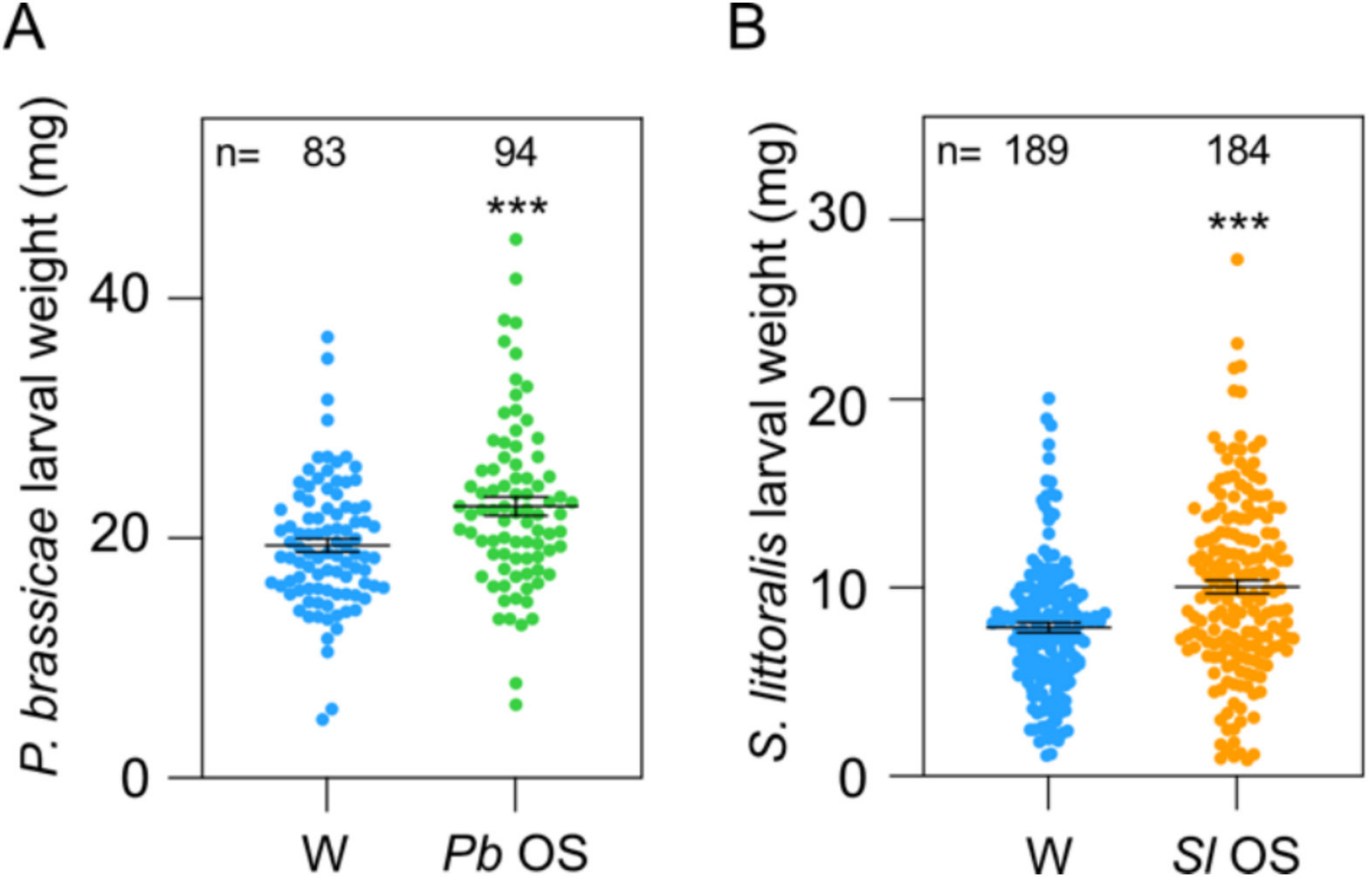
Performance of ***P. brassicae*** (*Pb*) and ***S. littoralis*** (*Sl*) larvae on OS‐pretreated plants. Neonate larvae were placed on 5‐week‐old plants that were wounded (W) or treated with insect OS. *P. brassicae* (A) and ***S. littoralis*** (B) larvae were weighed after 10 and 12 days, respectively. Means ± SE of three independent biological replicates are shown. Asterisks denote statistical differences between W and OS treatment (Student's *t*‐test, *** ***p*** < 0.001); *n*, number of larvae.

Having identified several defense‐related pathways that are either amplified/induced or suppressed/downregulated by *P. brassicae* or *S. littoralis* OS, we further asked whether OS application to a wound site decreases or increases of larval performance. To test this, we pretreated Arabidopsis leaves by wounding and applying water or wounding and applying OS. We then challenged the plants with *Pieris rapae* or *S. littoralis* larvae. Strikingly, caterpillars of both species gained significantly more weight when fed on plants pretreated with their respective OS, when compared to plants that were only wounded (Figure 9). The OS‐mediated increase in performance was more pronounced for *S. littoralis* than for *P. brassicae* larvae, which gained respectively about 30% and 10% more weight on average. These data are in line with our previous observation that OS application enhances performance of *S. littoralis* larvae and, interestingly, extends it to the adapted herbivore *P. brassicae* (Consales et al. 2012).

Since *S. littoralis* , but not *P. brassicae* , is susceptible to GS (Smallegange et al. 2007), the better performance of the non‐adapted insect could be the consequence of OS‐mediated downregulation of aliphatic GS biosynthesis discussed above. The observation that OS of *P. brassicae* , which is a specialist that even prefers to feed on plants rich in GS (Schweizer, Fernández‐Calvo, et al. 2013), also contained a suppressing activity suggests that OS may have additional defense targets besides the suppression of aliphatic GS biosynthesis. Alternatively, *P. brassicae* may still benefit from a reduced GS accumulation because the NSP protein that has evolved in this adapted species does not completely eliminate the toxic effect of GS (Wheat et al. 2007). Regardless, the effector activity associated with the OS of either species improved the fitness of their larvae, likely by negatively interfering with plant defenses. This result indicates that the negative effect of OS‐derived effectors on plant defense is likely stronger than the positive effect of OS‐derived HAMPs.

## 3 Discussion

Our analysis of wounded Arabidopsis leaves highlights the significant reprogramming of the transcriptome, primarily characterized by the activation of the JA pathway in combination with other hormonal pathways (Reymond et al. 2000; Ikeuchi et al. 2017; Kaur et al. 2024). Wounding alone results in the activation of plant defenses such as GS and flavonoid biosynthesis, ROS production, and other downstream immune responses. These results are in line with the established knowledge that JA is the central regulator of wound responses (Howe et al. 2018). Because we chose to apply a moderate wound treatment by piercing four tiny holes in three leaves per plant without harming the midvein, we certainly have not captured the whole wound‐induced transcriptome that has been previously identified by severely crushing leaves with forceps or applying more extensive wounding (Kimberlin et al. 2021; Chen et al. 2025). The rationale of our experiment was rather to mimic moderate herbivory by young larvae and to concentrate our study on the effect of OS.

Previous research also corroborates our finding that herbivory specifically downregulates genes involved in photosynthesis and plant growth (Ralph et al. 2006; Bilgin et al. 2010; Guo et al. 2018; Steinbrenner et al. 2022). The suppression of these processes is known to be part of the trade‐off between growth and defense (Guo et al. 2018). However, we show that application of caterpillar OS on wound sites has a substantial and additional effect by amplifying responses triggered by wounding and specifically inducing other plant defense processes. How OS‐derived factors amplify the wound response at the molecular level is unknown and would clearly deserve further investigation. There are already many known HAMPs from various arthropods (Stahl et al. 2018; Snoeck et al. 2022; Prajapati et al. 2024) but defining which and how these components target wound signaling may prove difficult. In addition, the variety of responses specifically induced by OS most likely underpin the presence of multiple HAMPs with different targets and mode of action.

Besides activation of the JA pathway, the OS‐mediated induction of other hormonal pathways suggests that perception of herbivory through HAMPs induces a broader hormonal crosstalk. These findings highlight the complexity of hormonal signaling wiring in plants and raise the question of whether the OS‐mediated activation of these pathways could be driven by effector activity. Salivary effector Bt56 of the whitefly *Bemisia tabaci* , for example, was shown to elicit the SA signaling pathway in maize in order to dampen plant JA signaling and facilitate the performance of the insect (Xu et al. 2019). We observed that OS of both insects trigger the expression of genes related to responses to pathogens and to systemic acquired resistance, which suggests the presence of microorganisms in the OS. Symbiotic bacteria are commonly present in insect OS, and some insect species exploit these microorganisms to induce SA responses in order to suppress anti‐herbivore JA‐mediated responses (Gimenez‐Ibanez and Solano 2013). OS from the Colorado potato beetle, for instance, was found to contain different bacteria that trigger an increase of SA levels, leading to a decreased accumulation of JA in response to herbivory (Chung et al. 2013). Although we found that OS induce the expression of genes related to microbial responses, it did not seem to influence the JA pathway to an extent that could indicate a significant impact of OS‐derived bacteria on plant defense via SA/JA crosstalk. To verify the contribution of OS‐derived microorganisms to transcriptional responses, we could feed the caterpillars with an antibiotic‐rich diet and assess the ability of the OS of such insects to induce immunity. However, the observation that chitin‐responsive genes are induced by OS indicates the presence of chitin either originating from the insect exoskeleton or from the peritrophic matrix (Lehane 1997; Zhu et al. 2016). Indeed, a recent study demonstrated that rice plants recognize herbivory via the chitin receptors CERK1 and CEBiP and that OS contain chitooligosaccharides that act as HAMPs (Kanda et al. 2025). In addition, it was reported that the herbivore‐induced flavonoid sakuranetin protects rice from planthopper by inhibiting the survival of fungal endosymbionts (Liu et al. 2023), suggesting that fungi may also be the source of chitin in OS. These findings clearly illustrate the need to consider herbivore attack as a complex biotic stress that may include additional microbial components.

In response to herbivory, a well‐known indirect defense response is the attraction of parasitoids by the release of herbivore‐induced plant volatiles (HIPVs). This phenomenon is primarily due to the perception of HAMPs, which in this case are fatty acid amino acid conjugates (FACs) identified in different leaf‐chewing herbivore species. FACs activate genes involved in the biosynthesis of oxygenated fatty acids, terpenes, methyl salicylate, and indole (Pare and Tumlinson 1999; Turlings and Erb 2018). Strikingly, none of these GO categories was enriched in the transcriptomes of wounded or OS‐treated plants. Arabidopsis Col‐0 ecotype is not particularly known for having strong indirect defenses although some attractiveness has been reported (Van Poecke and Dicke 2004). This apparent discrepancy should fuel further work on the role of OS in indirect defenses in Arabidopsis. However, there is evidence that some effectors prevent green leaf volatile emission by directly modifying or trapping these molecules (Jones et al. 2022). In this case, there is no effect on transcriptional regulation suggesting that other effectors may also suppress defense by acting directly on the functionality of defense molecules.

As insect OS are known to contain effectors that inhibit defenses to the benefit of larval growth (Prajapati et al. 2024), we focused our analysis on transcriptional changes that may indicate an OS‐mediated interference with plant defense. A striking finding is the OS‐mediated downregulation of aliphatic GS biosynthesis genes. Aliphatic GS constitute 70% of the total GS in the plant and a lack in their production renders the plant more susceptible to herbivory (Beekwilder et al. 2008; Müller et al. 2010). In this study, we show consistent OS‐mediated suppression of *MYC4*, *MYB28*, *MYB29*, *MYB76*, as well as general suppression of aliphatic GS biosynthesis genes. Given the crucial role of GS in Arabidopsis anti‐herbivore defenses, the inhibition of aliphatic GS production should provide a clear advantage to the generalist *S. littoralis* . Interestingly, we show that even the specialist *P. brassicae* , which prefers feeding on GS‐rich tissues, reduces the expression of genes involved in aliphatic GS production. This could indicate that the OS‐derived effector suppressing aliphatic GS biosynthesis may have evolved in the *Lepidoptera* clade before *P. brassicae* adapted to feed on GS‐rich tissues. Alternatively, since NSP does not fully abolish GS toxicity, this specialist insect may still benefit from lowering GS levels. Further research should aim at identifying the OS component and specific mechanism that leads to the suppression of aliphatic GS biosynthesis. One hypothesis could be that an effector inhibits the activity of a factor controlling the expression of aliphatic GS‐related *MYB*s. Intriguingly, FACs have also been postulated to suppress plant defenses by triggering ROS that inhibit the JA pathway (Block et al. 2018). Volicitin is a conjugate of hydroxylated linolenic acid and glutamine, and is found in several insect species including *S. littoralis* (Pohnert et al. 1999). The presence of FACs in *S. littoralis* and, potentially, *P. brassicae* OS may thus contribute to the observed downregulation of GS‐related genes. However, we previously showed that OS from *S. littoralis* and *P. brassicae* larvae reared on Arabidopsis mutants unable to produce fatty acids needed for FACs production were still able to suppress *ERF114* expression. In addition, treatment with FACs also did not suppress *ERF114* expression (Consales et al. 2012). Further studies should anyhow investigate the role of FACs in GS biosynthesis.

Although Arabidopsis eventually accumulates GS after several hours of herbivory and OS treatment induced transcription of indole‐GS genes (Schweizer, Fernández‐Calvo, et al. 2013), the quantitative contribution of OS suppression on the overall GS levels is unknown and will be only defined when effector(s) responsible for this effect are identified and their role validated by generating knocked‐out insects. Anyhow, these results highlight the antagonistic action of HAMPs and effectors in OS that may result from the long‐lasting coevolution between plants and insects.

ERF114 was already identified in a previous study from our group that looked at OS‐mediated suppression of gene expression with Arabidopsis DNA microarrays (Consales et al. 2012). ERF114 has been reported to participate in wound healing processes together with its homolog ERF115. Indeed, an *erf114erf115* double mutant displays a compromised callus formation after wounding (Zhang et al. 2022), indicating that the OS‐mediated suppression of ERF114 could compromise the ability of the plant to effectively heal the wounds inflicted by the herbivore. In addition to *ERF114* suppression, OS interfere with the expression of two wound healing regulators of the DOF family and two out of three members of the cytokinin‐dependent CYCD3 family. A quadruple *dof* mutant has reduced wound healing abilities and *CYCD3;1‐3* triple mutant is impaired in wound‐induced callus formation and, accounting for reduced callus formation, tissue attachment and pectin methyl esterification in response to wounding (Ikeuchi et al. 2017; Zhang et al. 2022). Thus, collectively, OS‐mediated suppression of wound healing genes may compromise the plant ability to recover from herbivore‐inflicted damage and allow improved feeding, along with the suppression of aliphatic‐GS biosynthesis, as illustrated by our finding of the enhanced performance of *P. brassicae* and *S. littoralis* larvae on OS‐treated plants.

Interestingly, ERF114 has also been shown to regulate lignin production during pathogenesis (Li et al. 2022). This may also contribute to the observed downregulation of genes participating in cell wall strengthening. Further analysis on the role of ERF114 during wounding might unveil the precise genes and pathways that are under the transcriptional control of this factor. Furthermore, how OS‐derived effectors inhibit its expression should be addressed. Also, it would be interesting to test if Arabidopsis lines overexpressing *ERF114* are more resistant to *S. littoralis* and *P. brassicae* herbivory.

The findings presented in this study give new insights into the mechanisms of effector‐mediated suppression of plant defenses in two lepidopteran herbivores. The ability of OS to potentially modulate the accumulation of defense compounds, cell wall strengthening, and wound healing mechanisms reflects the complexity of the strategies employed by insects to overcome plant defenses. Furthermore, the shared transcriptional responses observed in plants treated with *P. brassicae* and *S. littoralis* OS suggests that these two species employ similar strategies to suppress plant defenses, indicating the presence of conserved effectors in lepidopteran OS. Considering that in our experimental setup OS were applied once on the wounding site, while upon natural herbivory caterpillars continuously release OS on the leaf surface, we assume that the transcriptional effects of OS that we observe at 3 h would persist for a longer period. Thus, the activity of such effectors may give the insect a significant physiological advantage.

While this study provides valuable insights into the responses impaired by OS, further experiments are needed to identify the effector(s) responsible for the suppression of the plant defenses. Like with HAMPs, given the variety of affected processes, one must assume that *S. littoralis* and *P. brassicae* OS contain multiple effectors with diverse activities and targets. This is supported by the diversity of effectors already known in different plant–insect interactions (Stahl et al. 2018; Prajapati et al. 2024). For example, *Helicoverpa armigera* derived HARP1 and HAS1 were shown to interfere with the initiation of herbivore induced JA‐mediated transcriptional reprogramming, causing an impairment of the plant immune responses (Chen et al. 2019; Chen et al. 2023). Interestingly, homologs of HARP1 and HAS1 have been recently identified in *S. littoralis* and *S. exigua* OS proteomes (García‐Marín et al. 2025). The activity‐guided purification of *S. littoralis* and *P. brassicae* OS combined with targeted analysis of effector candidates from *S. littoralis* OS proteome might provide a successful strategy for the identification effectors. This would allow to further investigate their mechanism of action and validate their importance by gene knock‐out in insect hosts.

In conclusion, this study contributes to a better knowledge on the substantial and specific contribution of insect OS to plant defenses in Arabidopsis. Results highlight a complex interaction between plants and chewing herbivores at the wound site and uncover responses that are targeted by OS‐derived insect effectors, increasing the limited knowledge available up to date on this topic and providing hypotheses on how *P. brassicae* and *S. littoralis* may enhance their performance on host plants.

## 4 Experimental Procedures

### 4.1 Plant and Insect Growth Condition

*Arabidopsis thaliana* Col‐0 ecotype was sown in moist potting compost. Seeds were stratified on soil for 2 days at 4°C in the absence of light and then transferred to a growth chamber in short day conditions (22°C, 65% relative humidity, 10 h light, 100 μmol m^−1^ s^−1^). Ten days after germination plants were transferred to single pots.

*Spodoptera littoralis* eggs obtained from Syngenta (Stein, Switzerland) were stored at 10°C until use. For hatching, eggs were placed in an incubator at 28°C in a beaker containing a piece of moist tissue and covered with parafilm. After hatching, larvae were placed on Arabidopsis in a transparent plastic box in a growth chamber in short day conditions. *P. brassicae* was reared in a greenhouse on *Brassica oleracea* plants (Reymond et al. 2000). One day before collecting oral secretions (OS), *P. brassicae* larvae were transferred on Arabidopsis. OS were collected from fourth to fifth instar larvae by gently squeezing them and placing a pipette tip at their mouth. OS were subsequently stored at −20°C.

### 4.2 Plant Treatment

Five weeks after germination, plants were separated in groups of two per treatment. Three leaves of each plant, except for the unwounded controls, were wounded by piercing four holes of 1 mm each, avoiding the midvein. Two microliters of either H_2_O, *P. brassicae* OS, or *S. littoralis* OS were applied to each hole. After the treatment, plants were kept in the growth room chamber in short day conditions. Four independent biological replicates were carried out using the same procedure. Samples were collected either 3 or 24 h after wounding. Unwounded controls were collected 3 h after wounding. Leaf discs of 1 cm diameter around the wound site were collected, frozen in liquid nitrogen and stored at −80°C.

### 4.3 RNA Extraction, cDNA Synthesis, and qPCR

Leaf discs were ground in a Qiagen tissuelyser and RNA was extracted using a ReliaPrep RNA Tissue Miniprep System (Promega). To monitor gene expression by quantitative real‐time PCR (qPCR), reverse transcription was performed using M‐MLV retrotranscriptase (Invitrogen) for every sample in triplicates, starting with 500 mg of RNA in a final volume of 15 μL according to the manufacturer instructions. qPCR analysis was done by mixing 2 μL of cDNA, 0.2 μM of each primer (Table S3), 0.03 μM of reference dye and 10 μL of Brilliant III Ultra Fast SYBR Green qPCR Master Mix (Agilent) in a final volume of 20 μL. The reaction was performed in a QuantStudio 3 Real‐Time PCR System (Invitrogen) with the following program: 95°C for 3 min, 40 cycles of 10 s at 95°C and 20 s at 60°C. Relative mRNA abundance was normalized to the housekeeping gene SAND (At2g28390).

### 4.4 RNA Sequencing and Data Processing

Individual libraries made from the 150 ng of RNA of each sample were processed by the Genomic Technologies Facility (GTF) at the University of Lausanne. Samples were single‐end (SE) sequenced on an Illumina NovaSeq6000 generating reads with a length of 150 bp. Adapter‐free purity‐filtered reads were generated with Cutadapt (v. 2.5) (Martin 2011). Reads matching to ribosomal RNA sequences were removed with fastq_screen (v. 0.11.1). Remaining reads were further filtered for low complexity with reaper (v. 15‐065) (Davis et al. 2013). Reads were aligned against *A. thaliana* TAIR10.39 genome using STAR (v. 2.5.3a) (Dobin et al. 2013). The number of read counts per gene locus was summarized with htseq‐count (v. 0.9.1) (Anders et al. 2015) using gene annotation. Quality of the RNA‐seq data alignment was assessed using RSeQC (v. 2.3.7) (Wang et al. 2012). The htseq‐generated counts data was used for the analysis.

### 4.5 Data Normalization

Genes with low counts were filtered out according to the rule of one count per million (cpm) in at least one sample. Library sizes were scaled using TMM normalization. Subsequently, the normalized counts were transformed to cpm values and a log_2_ transformation was applied with the parameter setting prior. Counts = 1 (EdgeR v 3.30.3) (Robinson et al. 2010)). After data normalization, a quality control analysis was performed through samples correlation/clustering, UMAPs and principal components analysis (PCA). The sample treated with wounding and H_2_O of one of the batches was excluded because it was an outlier. Clustering and correlation plots showed some bias that seemed to link the replicates two‐by‐two (Replicates 1 and 2 clustered together and 2 and 4 clustered together but away from Replicates 1 and 2, especially in the samples collected at 24 h). This is likely due to the fact that batches of plants 1 and 2 had 2 weeks overlap in the growth room, as well as batches 3 and 4. We thus applied a batch‐correction (based on the replicates number, hence four batches, applied to both 3 and 24 h time points) (Ritchie et al. 2015).

### 4.6 Data Analysis

Differential expression for batch‐corrected scores was computed with the R Bioconductor package limma by fitting data to a linear model. Batches were directly integrated to the limma design matrix. To obtain DEGs, contrasts (control vs. wounding or treatments; wounding vs. treatments) were first summarized separately using moderated *t*‐statistics via the topTable limma function. *p*‐Values were adjusted using the Benjamini–Hochberg (BH) method, which controls for the false discovery rate (FDR).

A PANTHER classification system and the topGO R package was used to analyze all DEGs. A Fisher's exact test was applied with a cutoff of *p* < 0.05. For DEG subsets, GO term enrichment analysis was conducted using ShinyGO (Ge et al. 2020).

### 4.7 Insect Performance Assay

To monitor insect performance, 30–40 freshly hatched *P. brassicae* or 70–80 freshly hatched *S. littoralis* larvae were placed on 11 5‐week‐old pretreated Arabidopsis plants in transparent plexiglass boxes. For the pretreatment, three leaves of each plant were wounded by piercing four holes of 1 mm each, avoiding the midvein and 2 μL of either H_2_O, *P. brassicae* OS, or *S. littoralis* OS were applied to each hole. Plants were replaced every second day with freshly treated ones. *P. brassicae* and *S. littoralis* larvae were feeding on treated plants for respectively 10 and 12 days. Individual larval weights were determined on a high precision balance (Mettler‐Toledo; XP205DR, Switzerland).

## 5 List of Genes Mentioned in This Study

*ABCG6* (At5g13580), *ABCG16* (At3g55090), *AGP31* (At1g28290), *ARF3* (At2g33860), *ARR5* (At3g48100), *ARR8* (At2g41310), *ARR9* (At3g57040), *ARR11* (At1g67710), *AUX1* (At2g38120), *CSLD5* (At1g02730), *CHI* (At2g43590), *CYCD3;1* (At4g34160), *CYCD3;3* (At3g50070), *CYCD6–1* (At4g03270), *CYP79F1* (At1g16410), *CYP79F2* (A11g16400), *DIR23* (At2g21100), *DOF6* (At3g45610), *ERF109* (At4g34410), *ERF113* (At5g13330), *ERF114* (At5g61890), *ERF115* (At5g07310), *FAR5* (At3g44550), *HCA2*/*DOF5.6* (At5g62940), *IAA14* (At4g14550), *LAC5* (At2g40370), *LAC6* (At2g46570), *LECa* (At3g16530), *LECb* (At3g15356), *LOX4* (At1g72520), *LTP2* (At2g38530), *LTPG15* (At2g48130), *LTPG20* (At3g22620), *ML3* (At5g23820), *MYB28* (At5g61420), *MYB29* (At5g07690), *MYB41* (At4g28110), *MYB76* (At5G07700), *MYC2* (At1g32640), *MYC3* (At5g46760), *MYC4* (At4g17880), *PAE10* (At5g26670), *PEP1* (At5g64900), *PER33* (At3g49110), *PIN5* (At5g16530), *PLL18* (At3g27400), *RWP1* (At5g41040), *SHN3* (At5g25390), *TBL15* (At2g37720), *UGT73B5* (At2g15480), *WIND1* (At1g78280), *WIND2* (At1g22190), *WOX4* (At1g46480), *WOX5* (At3g12260), *WSD1* (At5g37300), *XTH31* (At3g44990).

## Author Contributions

Conceptualization: A.F.M., P.R.; Investigation: A.F.M.; Writing – review and editing: A.F.M., P.R.; Funding acquisition: P.R.

## Conflicts of Interest

The authors declare no conflicts of interest.

## Peer Review

The peer review history for this article is available in the Supporting Information for this article.

## Supporting information


**Data S1** Supplementary Peer Review.

**Table S1** Expression changes after wounding (W), application of *Pieris brassicae* oral secretions (Pb OS) or *Spodoptera littoralis* oral secretions (Sl OS). Values are the average of four biological replicates. CTL, untreated.

**Table S2** Expression changes after application of Spodoptera littoralis oral secretions (Sl OS) for 3 h or challenge with four–fifth instar Spodoptera littoralis larvae (Sl larvae) for 5 h. Values are the average of four biological replicates. Sl OS, RNAseq data, this study. Herbivory, microarray data from Schweizer et al., 2013.

**Table S3** List of primers used for qPCR.
**Figure S1.** Volcano plots of Arabidopsis gene expression in response to different treatments. Wounding (A, D), treatment with *P. brassicae* oral secretions (*Pb* OS) (B, E) and treatment with *S. littoralis* oral secretions (*Sl* OS) (C, F). Colored dots display genes which are significantly up‐ or downregulated (log2FC > 1, adj*P* < 0.05) whereas gray dots display genes that are below the significance thresholds.
**Figure S2.** OS effect on wound‐repressed genes. Expression of genes significantly repressed by wounding (W) (log2FC < −1, adj*P* < 0.05) after 3 h (A, B) and 24 h (C, D) is shown and compared to treatment with *P. brassicae* OS (A, C) or *S. littoralis* OS (B, D). Light and dark blue dots represent genes equally repressed between wounding and OS treatments. Light green or orange dots represent genes significantly more or less repressed by OS treatments than by W.
**Figure S3.** Specific gene downregulation by *P. brassicae* and *S. littoralis* OS. Genes significantly repressed by OS (log2FC < −1, adj*P* < 0.05) and not repressed by wounding (log2FC > −1 or log2FC < −1 but adj*P* > 0.05) after 3 h (A) and 24 h (B) are shown.
**Figure S4.** OS application mimics natural herbivory. Gene expression upon treatment with *S. littoralis* OS is compared to gene expression upon herbivory by *S. littoralis larvae*. Purple dots represent genes significantly induced in both experiments (log2FC > 0.58, adj*P* < 0.05). Gray dots represent genes not significantly induced in both experiments.
**Figure S5.** OS effect on JA‐related genes. Heatmap showing the expression profile of genes involved in JA biosynthesis, signaling and response. CPM, counts per million of reads.
**Figure S6.** qPCR validation of OS‐mediated suppression of wound‐induced *ERF114*. Relative expression of *ERF114* (At5g61890) (normalized to the housekeeping gene *SAND* (At2g28390)) represents means ± SE of three technical replicates. The experiments were repeated three times with similar results. Different letters represent significant differences at *p* < 0.05 (ANOVA, followed by Tukey HSD for multiple comparisons).
**Figure S7.** OS effect on wound healing genes. Heatmap showing the expression profile of (A) regulators of plant regeneration and suberin/cuticle accumulation, and (B) cytokinin, auxin and cyclin genes involved in plant regeneration. CPM, counts per million of reads.

## Data Availability

RNAseq data have deposited in the National Center for Biotechnology Information's Gene Expression Omnibus (GEO) under the accession number GSE288493.
