# Supplementary material for: Impact of Lepidopteran Oral Secretions on the Transcriptome of Arabidopsis thaliana
Source: Plant Direct. 2025 Jun 19;9(6):e70085. doi: 10.1002/pld3.70085 (PMC12178948; doi:10.1002/pld3.70085)
Supplement: Supplementary file 4 — Table S3 List of primers used for qPCR. Figure S1. Volcano plots of Arabidopsis gene expression in response to different treatments. Wounding (A, D), treatment with P. brassicae oral secretions (Pb OS) (B, E) and treatment with S. littoralis oral secretions (Sl OS) (C, F). Colored dots display genes which are significantly up‐ or downregulated (log2FC > 1, adjP < 0.05) whereas gray dots display genes that are below the significance thresholds. Figure S2. OS effect on wound‐repressed genes. Expression of genes significantly repressed by wounding (W) (log2FC < −1, adjP < 0.05) after 3 h (A, B) and 24 h (C, D) is shown and compared to treatment with P. brassicae OS (A, C) or S. littoralis OS (B, D). Light and dark blue dots represent genes equally repressed between wounding and OS treatments. Light green or orange dots represent genes significantly more or less repressed by OS treatments than by W. Figure S3. Specific gene downregulation by P. brassicae and S. littoralis OS. Genes significantly repressed by OS (log2FC < −1, adjP < 0.05) and not repressed by wounding (log2FC > −1 or log2FC < −1 but adjP > 0.05) after 3 h (A) and 24 h (B) are shown. Figure S4. OS application mimics natural herbivory. Gene expression upon treatment with S. littoralis OS is compared to gene expression upon herbivory by S. littoralis larvae. Purple dots represent genes significantly induced in both experiments (log2FC > 0.58, adjP < 0.05). Gray dots represent genes not significantly induced in both experiments. Figure S5. OS effect on JA‐related genes. Heatmap showing the expression profile of genes involved in JA biosynthesis, signaling and response. CPM, counts per million of reads. Figure S6. qPCR validation of OS‐mediated suppression of wound‐induced ERF114. Relative expression of ERF114 (At5g61890) (normalized to the housekeeping gene SAND (At2g28390)) represents means ± SE of three technical replicates. The experiments were repeated three times with similar results. Different letters [file PLD3-9-e70085-s001.pdf]

SUPPORTING INFORMATION

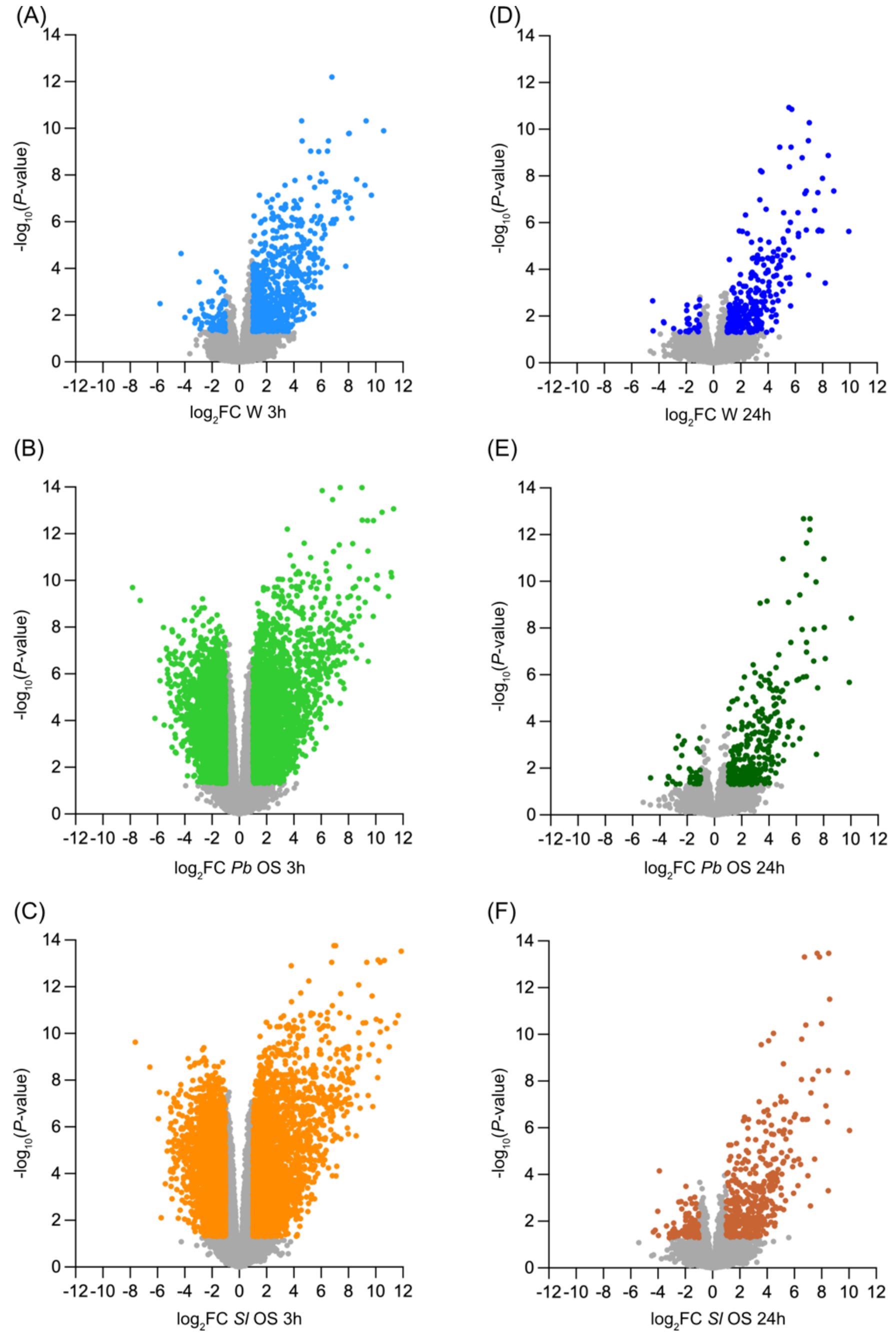

Figure S1. Volcano plots of Arabidopsis gene expression in response to different treatments. Wounding (A, D), treatment with *P. brassicae* oral secretions (*Pb* OS) (B, E) and treatment with *S. littoralis* oral secretions (*Sl* OS) (C, F). Colored dots display genes which are significantly up- or downregulated ($log_2FC>1$, adj$P<0.05$) whereas grey dots display genes that are below the significance thresholds.

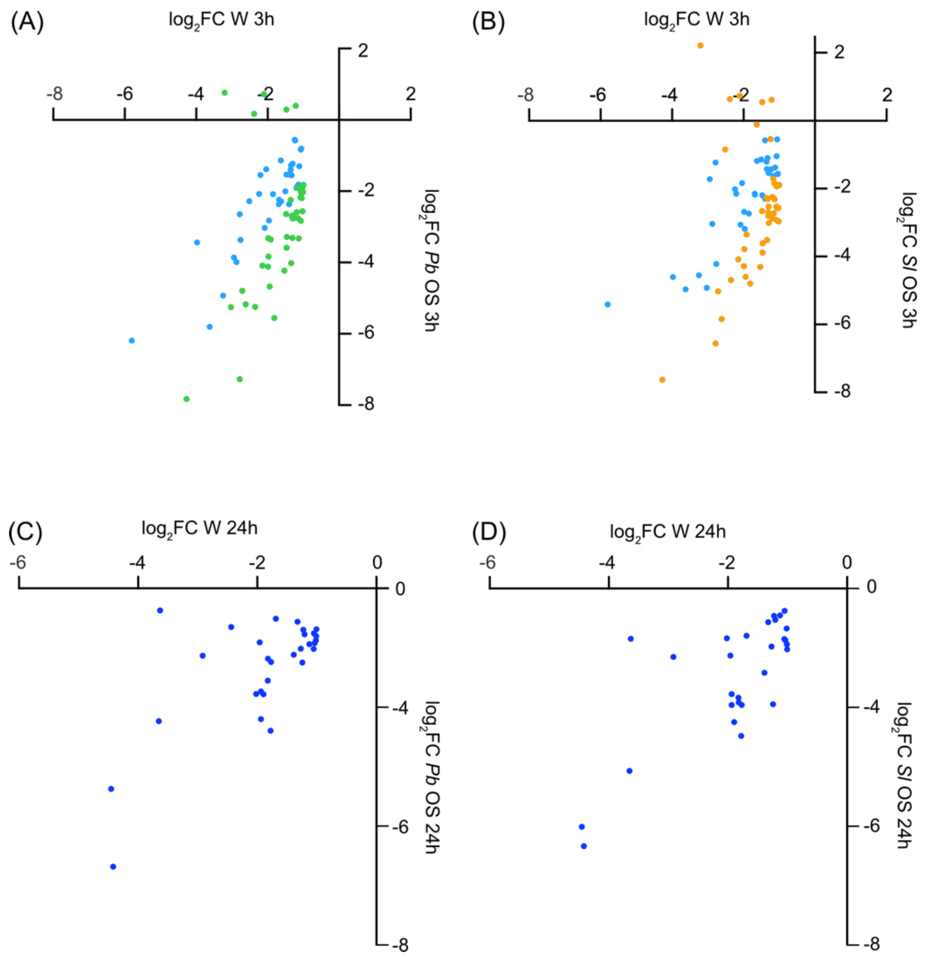

Figure S2. OS effect on wound-repressed genes.
Expression of genes significantly repressed by wounding (W) (log_2FC <-1, adj$P$<0.05) after 3 h (A, B) and 24 h (C, D) is shown and compared to treatment with *P. brassicae* OS (A, C) or *S. littoralis* OS (B, D). Light and dark blue dots represent genes equally repressed between wounding and OS treatments. Light green or orange dots represent genes significantly more or less repressed by OS treatments than by W.

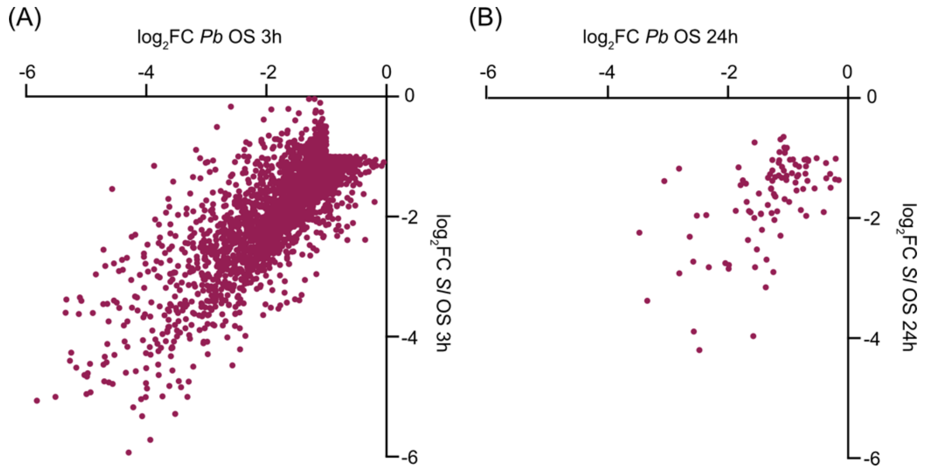

Figure S3. Specific gene downregulation by *P. brassicae* and *S. littoralis* OS.
Genes significantly repressed by OS ($log_2FC<-1$, adj$P<0.05$) and not repressed by wounding ($log_2FC>-1$ or $log_2FC<-1$ but adj$P>0.05$) after 3 h (A) and 24 h (B) are shown.

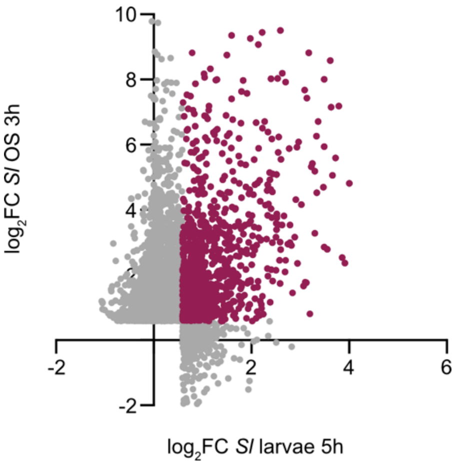

Figure S4. OS application mimics natural herbivory.
Gene expression upon treatment with *S. littoralis* OS is compared to gene expression upon herbivory by *S. littoralis larvae*. Purple dots represent genes significantly induced in both experiments ($log_2FC>0.58$, adjP<0.05). Grey dots represent genes not significantly induced in both experiments.

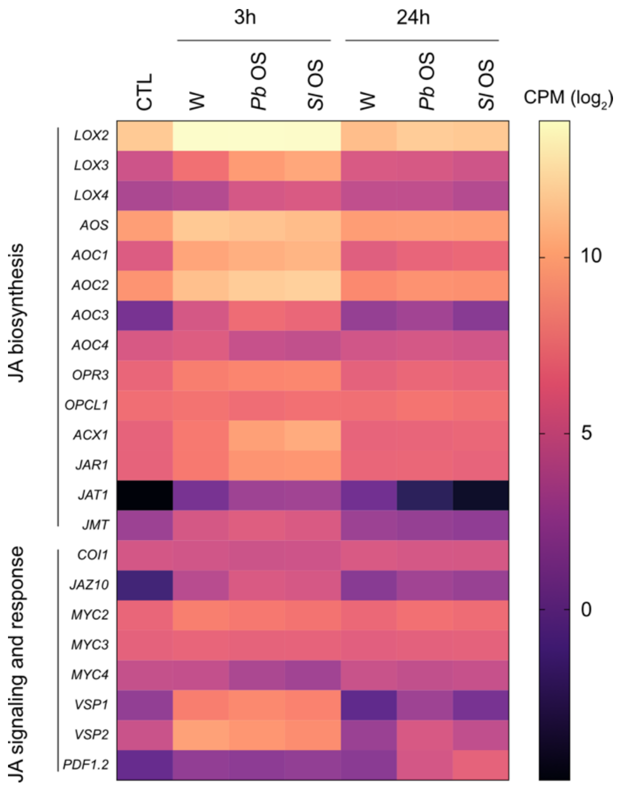

Figure S5. OS effect on JA-related genes.
Heatmap showing the expression profile of genes involved in JA biosynthesis, signaling and response. CPM, counts per million of reads.

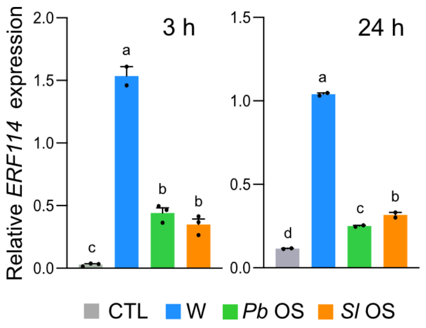

Figure S6. qPCR validation of OS-mediated suppression of wound-induced *ERF114*.
Relative expression of *ERF114* (At5g61890) (normalized to the housekeeping gene *SAND* (At2g28390)) represents means ± SE of three technical replicates. The experiments were repeated three times with similar results. Different letters represent significant differences at $P<0.05$ (ANOVA, followed by Tukey HSD for multiple comparisons).

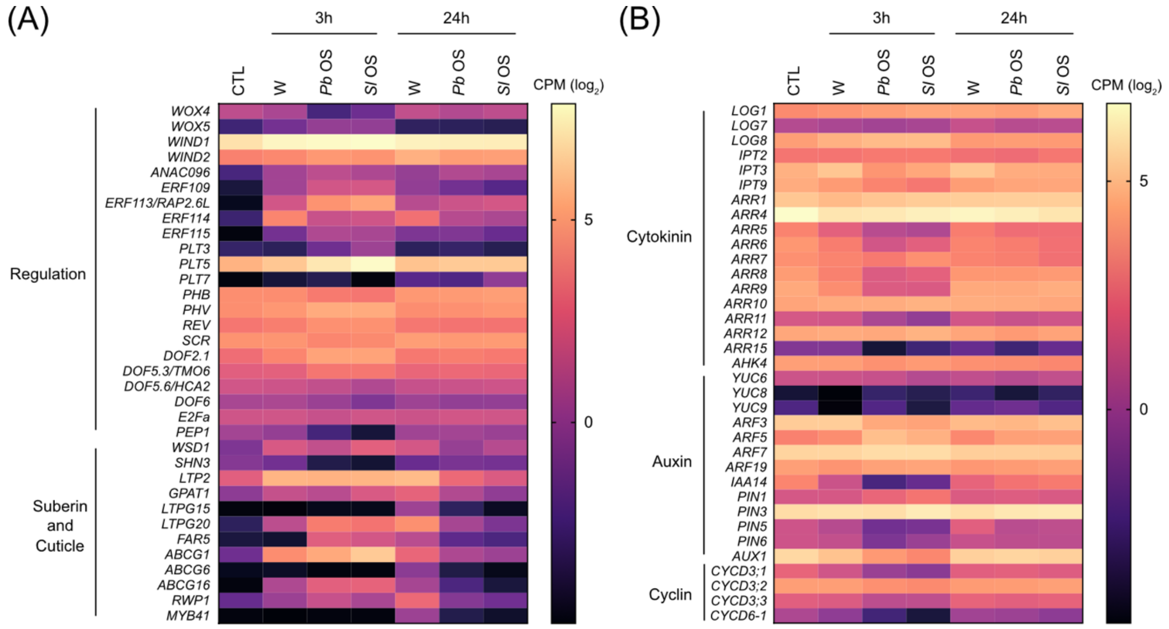

Figure S7. OS effect on wound healing genes.
Heatmap showing the expression profile of (A) regulators of plant regeneration and suberin/cuticle accumulation, and (B) cytokinin, auxin and cyclin genes involved in plant regeneration. CPM, counts per million of reads.

Table S3. List of primers used for qPCR.

Gene name	Gene ID	Primers ID	Sequences (5’-3’)
SAND	At2g28390	SAND-Fw	AACTCTATGCAGCATTTGATCCACT
		SAND-Rv	TGATTGCATATCTTTATCGCCATC
ERF114	At5g61890	ERF114-Fw	ATTACTCCTCCAACCAAATTCC
		ERF114-Rv	GTCTTCTGGTGGCTCTTCT
MYB28	At5g61420	MYB28-Fw	TCCCGATCGAGCTCAATGCCT
		MYB28-Rv	TGGAAGTGGCCTTAGCCGCAA
CYP79F1	At1g16410	CYP79F1-Fw	GTCGACGTGGCTGCATCGGT
		CYP79F1-Rv	GGTGCCAAGCGTGGCTCAAC
CYP79F2	At1g16400	CYP79F2-Fw	TGGGCTAGGCCGGAACCCTA
		CYP79F2-Rv	ACCGACGCAGCCACGTCTAC
