# Supplementary material for: Impact of Lepidopteran Oral Secretions on the Transcriptome of Arabidopsis thaliana
Source: Plant Direct. 2025 Jun 19;9(6):e70085. doi: 10.1002/pld3.70085 (PMC12178948; doi:10.1002/pld3.70085)
Supplement: Supplementary file 1 — Data S1 Supplementary Peer Review. [file PLD3-9-e70085-s004.pdf]

Plant Direct
Open Access

Impact of lepidopteran oral secretions on the transcriptome of Arabidopsis thaliana

Prof. Philippe Reymond, Dr. Angel Fernandez Martin

Decision letter round 1

May 23, 2025

Prof. Philippe Reymond

University of Lausanne

Plant Molecular Biology

Lausanne 1015

Switzerland

RE: Impact of lepidopteran oral secretions on the transcriptome of Arabidopsis thaliana

Dear Philippe:

Thank you for submitting to Plant Direct. All required reviews have been returned and we have now finished our evaluation of your manuscript. In light of the reviewers', further revisions are needed before the paper can be accepted for publication in Plant Direct.

Please view the reviewers' comments below and use their suggestions as a guide while you work on your revision. You'll see one reviewer is strongly critical - please ensure a full response, noting that we may send this out for re-review if necessary.

When uploading the revised version of this article, please be sure to include the following:

-A word document that contains your response to the reviewers. You should respond to each reviewer comment and note the changes made to the manuscript. If you do not agree with a reviewer's comment and choose not to make a suggested revision, please explain why. Please try to provide as complete an answer as possible to each reviewer's criticisms in the "response to reviewers" section.

-A tracked changes document

- A clean version of the latest version of the manuscript

To upload your revision, please click the link below.

https://plantdirect.msubmit.net/cgi-bin/main.plex?el=A7Lr1nt3A3ChS5I4A9ftdfLCTzpNwcPPke3qDozPPZAZ

In order to provide as timely a service as possible, we ask that your revision is resubmitted within three months after receipt of this request. If an extension is needed, please send a request, along with a brief explanation, to the editorial office at plantdirect@wiley.com .

Thank you very much for giving us an opportunity to review your work. I look forward to receiving the next version.

Best wishes,

Jonathan

Dr Jonathan Ingram

Supervising Editor

-- Reviewer comments:

Reviewer #1:

The manuscript reports a transcriptomic dataset of Arabidopsis plants comparing unwounded plants, wounded plants, and wound + OS treated plants with two species of Lepidopteran larval OS. Two timepoints are analyzed. Interesting genes are validated by qPCR analysis, and the suppressive effects of OS on host defenses are complemented with a separate pretreatment and larval growth assay. Methods and analyses are appropriate throughout. While specific OS components which elicit or suppress defense are not described, the manuscript will be a very useful dataset for both Arabidopsis and crop researchers interested in wound response, specialized metabolite biosynthesis, and herbivore-induced defenses.

Several notable findings include:

1. The manuscript highlights OS-suppressed genes, a category which is rarely emphasized in similar types of studies, but which are of increasing interest due to recent molecular characterization of Lepidopteran effector proteins. In particular the observation of particular TFs and PROPEP1 suppression is of general interest.

2. OS pretreatment increases larval growth of Pieris brassicae, meaning that suppression of defenses extends to several species when consuming Arabidopsis. It increasingly appears that Arabidopsis is particularly susceptible to Lepidoptera suppression mechanisms, perhaps due to insufficient host recognition mechanisms which would counteract OS suppressive functions.

3. The results and discussion sections are very well written and enlightening, especially in regard to wound healing, effectors from piercing/sucking herbivores, and GLS biosynthesis and function.

I only have minor suggestions:

- Figures which highlight particular genes analyzed by qPCR could provide gene IDs (ATxGxxxxx) in the legends to facilitate cross-referencing to the supplemental tables and other datasets

- A Spodoptera littoralis OS proteome was recently published and could be discussed, especially for the presence of REPAT proteins homologous to the characterized Helicoverpa armigera HARP1 and HAS1 effectors. https://link.springer.com/article/10.1007/s10886-025-01571-9

- Although specific elicitors and effectors are not measured here, the effects of the general OS component N-linolenoyl-L-glutamine on Arabidopsis have been described https://academic.oup.com/jxb/article/69/5/1235/4781664, and potentially may act as supressive molecules on Arabidopsis. If S.littoralis and P.brassicae fatty acid amino acid conjugates have been characterized (perhaps not?), the paper could discuss the potential role of these components on host signaling pathways.

Reviewer #2:

In this article, only appropriately 800 DEGs were identified between the untreated sample and the sample 3 hours post wounding treatment. And, the DEGs were even less (around 300), between the untreated sample and the sample 24 hours post wounding. In my opinion, this is impossible. Some genes that the author claimed were unchanged in expression in plants upon wounding, like LOX4, U73B5, CYP79F1 and CYP79F2, were significantly induced by wounding in our study and in some of the reported transcriptomes. Given that all the conclusions of this study are derived from transcriptome analysis, they may potentially be subject to certain inaccuracies.

Decision letter round 2

May 30, 2025

Plant Direct

Open Access

Prof. Philippe Reymond

University of Lausanne

Plant Molecular Biology

Lausanne 1015

Switzerland

MSID: 2025-01765-TWR1

MS TITLE: Impact of lepidopteran oral secretions on the transcriptome of Arabidopsis thaliana

Dear Philippe:

I am pleased to inform you that your manuscript "Impact of lepidopteran oral secretions on the transcriptome of Arabidopsis thaliana" has been accepted for publication in Plant Direct. Thank you for your careful response and the changes made.

Your article will appear online in the next available issue of Plant Direct. To ensure your article gets published as quickly as possible, please pay attention to the steps detailed below. We have found that most of the delays happen at this stage, especially at the payment stage, so please respond as quickly as possible when prompted.

License Agreement: Once your article has been accepted it will move to Production and undergo admin and file checking - you may receive an email with any queries we have at this stage. When all required items are received by the publisher and queries resolved, the corresponding author will receive an email from Wiley's Author Services system which will ask them to log on at https://authorservices.wiley.com/bauthor and will present them with the appropriate license for completion. Your article cannot be published until both the signed license agreement and payment of the article fee have been received.

Payment of the Open Access Article Publication Fee: All articles published in Plant Direct are fully open access: immediately and freely available to read, download and share. Plant Direct charges a publication fee to cover the publication costs. The corresponding author for this manuscript should have already received a quote from the payments team (cs-openaccess@wiley.com) with the estimated article publication fee; please email cs-openaccess@wiley.com if this has not been received. The corresponding author should log on to the Wiley Author Services site, where the publication fee can be paid by credit card or an invoice. Pro Forma can also be requested. Payment of the publication charge must be received before the article will be published online.

Proofs: You will have the opportunity to look over your paper once more when you receive the author proofs for your article. The proofs will be with you in approximately two weeks. Please note

that, in addition to publishing reviewer comments, the author's responses to review comments will also be published alongside the final version of the paper. If you would not like the author's responses to be published, please contact the editorial office at plantdirect@wiley.com .

Promotion of your article: You can help your research get the attention it deserves! Check out Wiley's free Promotion Guide for best-practice recommendations for promoting your work at www.wileyauthors.com/eeo/guide. And learn more about Wiley Editing Services which offers professional video, design, and writing services to create shareable video abstracts, infographics, conference posters, lay summaries, and research news stories for your research at www.wileyauthors.com/eeo/promotion.

Thank you again - really interesting work. If you have any questions, feel free to contact the editorial office at plantdirect@wiley.com .

Best wishes,

Jonathan

Dr Jonathan Ingram

Supervising Editor, Plant Direct

Response to reviewer round 1

Dear Dear Philippe:

Thank you for submitting to Plant Direct. All required reviews have been returned and we have now finished our evaluation of your manuscript. In light of the reviewers', further revisions are needed before the paper can be accepted for publication in Plant Direct.

Please view the reviewers' comments below and use their suggestions as a guide while you work on your revision. You'll see one reviewer is strongly critical - please ensure a full response, noting that we may send this out for re-review if necessary.

When uploading the revised version of this article, please be sure to include the following:

-A word document that contains your response to the reviewers. You should respond to each reviewer comment and note the changes made to the manuscript. If you do not agree with a reviewer's comment and choose not to make a suggested revision, please explain why. Please try to provide as complete an answer as possible to each reviewer's criticisms in the "response to

reviewers" section.

-A tracked changes document

- A clean version of the latest version of the manuscript

To upload your revision, please click the link below.

https://plantdirect.msubmit.net/cgibin/main.plex?el=A7Lr1nt3A3ChS5I4A9ftdfLCTzpNwcPPke3qDozPPZAZ

In order to provide as timely a service as possible, we ask that your revision is resubmitted within three months after receipt of this request. If an extension is needed, please send a request, along with a brief explanation, to the editorial office at plantdirect@wiley.com .

Thank you very much for giving us an opportunity to review your work. I look forward to receiving the next version.

Best wishes,

Jonathan

Dr Jonathan Ingram

Supervising Editor.

ANSWER: Thank you for the opportunity to revise our ms. We have addressed the reviewers comments as follows:

- for reviewer #1, we added the requested ATG numbers in Figure legends and mentioned the two suggested studies on S. littoralis OS proteome and suppressive role of FACs in the Discussion.

- for reviewer #2, we added some text to better explain that the experimental design was aimed to inflict moderate damage to Arabidopsis leaves, mimicking feeding by a few young larvae. This might explain the relatively lower number of wound-induced genes. We also specified that our goal was not to capture the whole wound-induced trancriptome but rather to evaluate the contribution of insect OS.

We include a revised vrsion with track changes and a clean version.

Reviewer comments:

Reviewer #1:

The manuscript reports a transcriptomic dataset of Arabidopsis plants comparing unwounded

plants, wounded plants, and wound + OS treated plants with two species of Lepidopteran larval OS. Two timepoints are analyzed. Interesting genes are validated by qPCR analysis, and the suppressive effects of OS on host defenses are complemented with a separate pretreatment and larval growth assay. Methods and analyses are appropriate throughout. While specific OS components which elicit or suppress defense are not described, the manuscript will be a very useful dataset for both Arabidopsis and crop researchers interested in wound response, specialized metabolite biosynthesis, and herbivore-induced defenses.
Several notable findings include:
1. The manuscript highlights OS-suppressed genes, a category which is rarely emphasized in similar types of studies, but which are of increasing interest due to recent molecular characterization of Lepidopteran effector proteins. In particular the observation of particular TFs and PROPEP1 suppression is of general interest.
2. OS pretreatment increases larval growth of Pieris brassicae, meaning that suppression of defenses extends to several species when consuming Arabidopsis. It increasingly appears that Arabidopsis is particularly susceptible to Lepidoptera suppression mechanisms, perhaps due to insufficient host recognition mechanisms which would counteract OS suppressive functions.
3. The results and discussion sections are very well written and enlightening, especially in regard to wound healing, effectors from piercing/sucking herbivores, and GLS biosynthesis and function.
ANSWER: We thank the reviewer for the positive feedback on our study.
I only have minor suggestions:
- Figures which highlight particular genes analyzed by qPCR could provide gene IDs (ATxGxxxxx) in the legends to facilitate cross-referencing to the supplemental tables and other datasets
ANSWER: We included the ATG numbers in the figure legends.
- A Spodoptera littoralis OS proteome was recently published and could be discussed, especially for the presence of REPAT proteins homologous to the characterized Helicoverpa armigera HARP1 and HAS1 effectors (https://link.springer.com/article/10.1007/s10886-025-01571-9)
ANSWER: Thank you for mentioning this interesting study. We mentioned this work in the Discussion (lines 618-626).

- Although specific elicitors and effectors are not measured here, the effects of the general OS component N-linolenoyl-L-glutamine on Arabidopsis have been described (https://academic.oup.com/jxb/article/69/5/1235/4781664), and potentially may act as supressive molecules on Arabidopsis. If S.littoralis and P.brassicae fatty acid amino acid conjugates have been characterized (perhaps not?), the paper could discuss the potential role of these components on host signaling pathways.

ANSWER: Thanks for this suggestion. We included some text to mention the role of FACs as HAMPs and their potential role as effectors (lines 531-535 and 561-570).

Reviewer #2 (Comments for the Author):

In this article, only appropriately 800 DEGs were identified between the untreated sample and the sample 3 hours post wounding treatment. And, the DEGs were even less (around 300), between the untreated sample and the sample 24 hours post wounding. In my opinion, this is impossible. Some genes that the author claimed were unchanged in expression in plants upon wounding, like LOX4, U73B5, CYP79F1 and CYP79F2, were significantly induced by wounding in our study and in some of the reported transcriptomes. Given that all the conclusions of this study are derived from transcriptome analysis, they may potentially be subject to certain inaccuracies.

ANSWER: Our study is based on four independent replicates and the expression of some interesting genes were validated by RT-QPCR. We thus believe that the reason for a seemingly low number of wound-induced genes has to be explained by the moderate wounding treatment that consisted of piercing four 1-mm holes in threee leaves. This contrasts with other transcriptome studies where severe crushing of leaves with forceps or more intense wounding were applied. We anyhow see induction of known wound-induced marker genes (LOX2, LOX3, VSP2, JAZ10, see Table S1).

We added some text to explain our experimental design and to mention that our aim was not to capture the whole wound-inducible transcriptome but to mimick moderate herbivory by young larvae (lines 133-135 and 484-489). We also stress that our goal was primarily to evaluate the specific contribution of insect OS, which we believe is demonstrated here.

Regarding specifically the expression of CYP79F1 and CYP79F2, we recently analyzed these genes in the frame of another project and found that they are induced after 4 h of wounding

(similar set-up as here), supporting the hypothesis that a moderate wound treatment is not strong enough to see induction of these genes at 3 h but that they might be upregulated later on (see Figure below).

Fig. X P. brassicae OS suppress the expression of wound-induced and GS biosynthesis genes. Plants were treated for 4 h with P. brassicae OS (Pb OS). Relative expression was monitored by RT-QPCR and normalized to the housekeeping gene SAND. Values are means ± SE of one representative experiment with technical triplicates. The experiment was repeated with similar results. Letters indicate significant differences at $P<0.05$ (ANOVA, followed by Tukey HSD for multiple comparisons). CTL, unwounded; W, wounding + H2O.
